# Pharmacological Fingerprints of Contextual Uncertainty

**DOI:** 10.1371/journal.pbio.1002575

**Published:** 2016-11-15

**Authors:** Louise Marshall, Christoph Mathys, Diane Ruge, Archy O. de Berker, Peter Dayan, Klaas E. Stephan, Sven Bestmann

**Affiliations:** 1 Sobell Department of Motor Neuroscience and Movement Disorders, Institute of Neurology, University College London, London, United Kingdom; 2 Wellcome Trust Centre for Neuroimaging, Institute of Neurology, University College London, London, United Kingdom; 3 Max Planck UCL Centre for Computational Psychiatry and Ageing Research, University College London, London, United Kingdom; 4 Department of Psychology and Neurosciences, Leibniz Research Centre for Working Environment and Human Factors, Technical University Dortmund, Dortmund, Germany; 5 Gatsby Computational Neuroscience Unit, University College London, London, United Kingdom; 6 Translational Neuromodeling Unit, Institute for Biomedical Engineering, University of Zurich & ETH Zurich, Zurich, Switzerland; 7 Max Planck Institute for Metabolism Research, Cologne, Germany; Oxford University, UNITED KINGDOM

## Abstract

Successful interaction with the environment requires flexible updating of our beliefs about the world. By estimating the likelihood of future events, it is possible to prepare appropriate actions in advance and execute fast, accurate motor responses. According to theoretical proposals, agents track the variability arising from changing environments by computing various forms of uncertainty. Several neuromodulators have been linked to uncertainty signalling, but comprehensive empirical characterisation of their relative contributions to perceptual belief updating, and to the selection of motor responses, is lacking. Here we assess the roles of noradrenaline, acetylcholine, and dopamine within a single, unified computational framework of uncertainty. Using pharmacological interventions in a sample of 128 healthy human volunteers and a hierarchical Bayesian learning model, we characterise the influences of noradrenergic, cholinergic, and dopaminergic receptor antagonism on individual computations of uncertainty during a probabilistic serial reaction time task. We propose that noradrenaline influences learning of uncertain events arising from unexpected changes in the environment. In contrast, acetylcholine balances attribution of uncertainty to chance fluctuations within an environmental context, defined by a stable set of probabilistic associations, or to gross environmental violations following a contextual switch. Dopamine supports the use of uncertainty representations to engender fast, adaptive responses.

## Abstract 2

Pharmacological interventions and hierarchical Bayesian modelling pinpoint the roles of noradrenaline, acetylcholine, and dopamine in computing different forms of uncertainty and in sensitizing actions to our beliefs about uncertainty.

## Author Summary

Interacting with dynamic and ever-changing environments requires frequent updating of our beliefs about the world. By learning the relationships that link events in the current environmental context, it is possible to prepare and execute fast, accurate responses to those events that are predictable. However, the world’s complex dynamics give rise to uncertainty about the relationships that exist between events and uncertainty about how these relationships might change over time. Several neuromodulators have been proposed to signal these different forms of uncertainty, but their relative contributions to updating beliefs and modulating responses have remained elusive. Here we combine a probabilistic reaction time task, pharmacological interventions, and a hierarchical Bayesian learning model to identify the roles of noradrenaline, acetylcholine, and dopamine in individual computations of uncertainty. We propose that noradrenaline modulates learning about the instability of the relationships that link environmental events. Acetylcholine balances the attribution of uncertainty to unexpected events occurring within an environmental context or to gross violations of our expectations following a context change. In contrast, dopamine sensitises our actions to our beliefs about uncertainty.

## Introduction

Adaptive performance in dynamic environments depends on our ability to represent and manipulate internal estimates of the world’s statistical structure [1–4]. By tracking the environment’s underlying regularities, an agent can learn the causes of its sensory input and thus the likelihood that a particular event will occur. In turn, this permits anticipatory action preparation and the rapid execution of responses [5].

However, the environment’s richly complicated sources of noise and latent structure present us with various forms of uncertainty. For instance, a London commuter predicting her journey time to work faces three distinct forms. First, there is irreducible uncertainty, which captures the randomness inherent in any complex environment and is undiminished by learning; an unplanned station closure could cause journey delays and thus influence the accuracy of the commuter’s estimated arrival time on any given day. Second, after moving to a new part of town, the duration of the commuter’s chosen route to work may be unclear, producing uncertainty about how likely she is to arrive at work on time. Over repeated journeys, this estimation uncertainty falls as the commuter learns about the probabilistic relationships within the current environmental context. For example, she learns to predict the frequent delays on this new route due to congestion during the morning rush hour, although these delays may vary with local dips and surges in the number of passengers using the service. Volatility uncertainty arises from our beliefs about the stability of the environment and thus how quickly probabilistic relationships are changing between contexts. A major sporting event, such as the London Olympics, may bring a large influx of additional passengers for an unknown period of time and with unexpected effects on transport performance, making it harder to predict future journey times until these changes have been taken into account.

### The Brain Computes Different Forms of Uncertainty

An assortment of theoretical, behavioural, and neurobiological evidence suggests that the brain computes uncertainty estimates relating to the environment’s sensory events, contextual associations, and their changes over time [6–17]. Uncertainty about the validity of one’s own perceptual beliefs about the world should have the effect of suppressing top-down prior expectations relative to new bottom-up sensory evidence, promoting learning about the current environmental context [18]. With their broad distribution and extensive connectivity, neuromodulatory networks are well placed to facilitate the widespread changes in gain required to support such a function [19,20]. In particular, acetylcholine (ACh) and noradrenaline (NA) are known to enhance bottom-up, feedforward thalamocortical transmission of sensory information relative to top-down, intracortical, and feedback processing [4,21–30].

#### Proposed roles for NA and ACh in computations of uncertainty

Within a stable environmental context, uncertainty arises from ignorance about, and the unreliability of, probabilistic cues predicting upcoming sensory events. Learning the environment’s underlying statistical regularities means that responses to explicitly and predictably cued events are typically faster than to those believed improbable [31–34]. This effect is modulated by pharmacological [35,36], surgical [37,38], and neurodegenerative [39] manipulations of ACh. For example, human cholinergic basal forebrain activity has been shown to reflect an individual’s uncertainty about probabilistic cue-outcome relationships [15]. More specifically, ACh appears to increase the rate at which humans learn probabilistic relationships under estimation uncertainty, supporting the idea that ACh enhances learning accorded to stimuli with uncertain predictive consequences [40] by suppressing the use of outdated top-down cues and boosting bottom-up sensory processing [4].

While NA plays no consistent role in probabilistic cueing [41,42], it is thought to offer an interrupt signal when volatility uncertainty arises between contexts [41–47]. Learning to make accurate predictions from the strongly unexpected observations that follow a contextual switch necessitates heightened sensory vigilance and a disregard for outdated top-down expectations. NA, with its broad neural network capable of triggering multiple, simultaneous changes across the brain [48], is well placed to rapidly coordinate this process. Indeed, neurons in the locus coeruleus (LC), the primary source of cortical NA, show strong responses to unexpected environmental changes [49,50]. Pharmacologically up-regulating NA accelerates the detection of unexpected switches in the predictive properties of sensory stimuli [51], while noradrenergic deafferentation of rat medial frontal cortex impairs behavioural adaptation to contextual switches [52]. Moreover, blood-oxygen-level-dependent (BOLD) activity in human LC has been shown to dynamically track volatility uncertainty [16], and pupil dilation—which is influenced by (nor)adrenergic afferents [53]—correlates with unexpected changes in probabilistic context [54,55].

### Motor Responses Are Sensitive to Uncertainty

Thus, uncertainty representations existing within and between environmental contexts are crucial for optimal predictions about the probability of future events. While good predictions facilitate anticipatory preparation of appropriate behavioural responses [5], they are not sufficient for adaptive performance in dynamic environments. An additional mechanism is required to modify action selection based on one’s own beliefs about the latent changes in the environment and/or the occurrence of unexpected events. Indeed, when an unexpected event occurs, humans are capable of engaging resources to inhibit a prepared response and replace it with an alternative [56,57], albeit at the expense of a prolonged reaction time (RT) [58,59].

#### Proposed role for dopamine in response modulation

There is considerable evidence linking the neuromodulator dopamine (DA) to flexible behaviour [60–64]. Dopaminergic deficits due to Parkinson’s disease are associated with specific flexibility impairments in both motor [59,65] and cognitive domains [60,66], with performance restored by dopaminergic medication [59,67]. In healthy individuals, pharmacological DA depletions impair adaptive reactions to unexpected events occurring within a broadly predictable context [58]. However, it remains unclear whether DA supports accurate response selection by facilitating perceptual belief updating [15] or by modulating the sensitivity of response selection to perceptual beliefs.

### A Unified Framework of Uncertainty

In sum, while physiological, pharmacological, behavioural, and theoretical work has suggested separable neuromodulatory involvement in different uncertainty computations, attempts to characterise the relative contributions of NA, ACh, and DA within a single computational scheme are lacking (but see noticeable exceptions from Varazzani et al. [68] contrasting the roles of NA and DA in motivation and Brown et al. [69] assessing NA and ACh involvement in orienting responses to environmental novelty). Here we employ a Hierarchical Gaussian Ffilter (HGF) model [9,10] to track human learning and response modulation in dynamic, probabilistic environments under pharmacological NA, ACh, and DA interventions.

The HGF has been successfully applied in several recent studies of learning under volatility [15,17,70–74]. We developed a novel instantiation of the HGF with two components: a three-level perceptual model of an agent’s mapping from environmental causes to sensory inputs and a response model that maps those inferred environmental causes to observed RT responses [75]. Thus, we sought to disentangle the effects of pharmacological interventions of the NA, ACh, and DA neuromodulatory systems on participant-specific perceptual belief updating from those on the sensitivity of motor responses to perceptual estimates.

## Results

**Fig 1:**
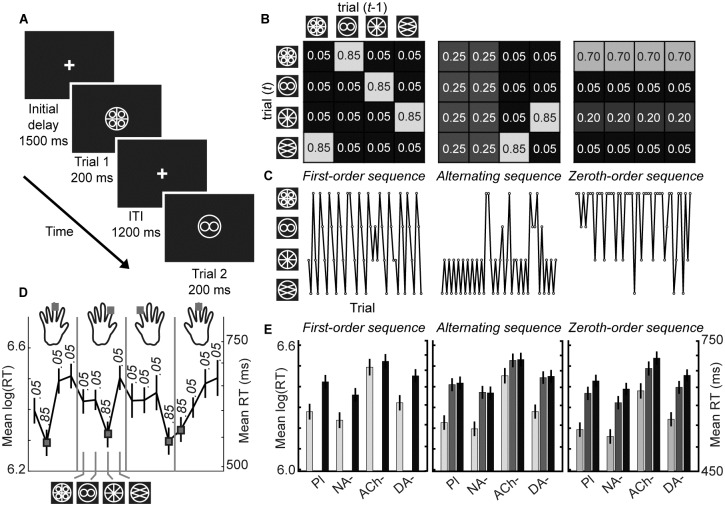
Task design. (A) Trial sequence. A trial began with the presentation of a central white fixation cross against a black background. After an initial delay of 1,500 ms at the start of each block, one of four visual stimuli was presented for 200 ms. Participants were required to make a speeded button-press response before the end of a 1,200 ms intertrial interval (ITI). (B) Stimulus transitions were generated by one of eight different transition matrices (TMs), which changed every 50 trials without explicit indication to the participant. These TMs comprised two different first-order sequences, two alternating sequences, and four zeroth-order sequences, each of which occurred three times in a pseudorandom order across 1,200 trials. The overall probability of each stimulus was equal across the 1,200 trials. For full details, see S1 Fig. (C) Example trial sequences generated from the three example TMs in 1B. (D) By tracking the transition probabilities, participants could learn to predict high probability events and prepare to make the correct button press accordingly. Faster responses were observed for predictable stimuli compared to unexpected stimuli. Here we depict Placebo group log(RTs) (mean ± standard error of the mean [SEM]) for each of the 16 possible combinations between consecutive stimuli for the first-order sequence shown in B. Grey boxes indicate stimulus combinations with a high transition probability. (E) Indeed, across all types of TM, responses were faster for stimuli with higher transition probabilities (mean ± SEM). http://dx.doi.org/10.6084/m9.figshare.3796314.v1.

According to a double-blind, between-subject design, 128 healthy participants undertook a serial probabilistic RT task (Fig 1) after having received a noradrenergic α1-receptor antagonist (prazosin; NA- group), a cholinergic M1-receptor antagonist (biperiden; ACh- group) or a dopaminergic D1/D2 receptor antagonist (haloperidol; DA- group), or a placebo. Data from 124 participants are reported. Four participants were excluded from analyses, three due to high missed response rates (≥11%) and one because the behavioural model parameter estimation (using the Broyden-Fletcher-Goldfarb-Shanno algorithm) did not converge. The four drug groups were matched for gender (Kruskal-Wallis test: H_3_ = 0.53, *p* = 0.912), age (one-way ANOVA: F_3,120_ = 0.46, *p* = 0.714), body weight (F_3,120_ = 2.24, *p* = 0.087), education level (H_3_ = 1.31, *p* = 0.727), and all baseline psychometric measures taken (Table 1).

**Table 1:** Participant details for each experimental group.

	Placebo (n = 32)	NA- (*n* = 31)	ACh- (*n* = 29)	DA- (*n* = 32)	Between-Group Difference?
**Gender (Number Male)**^#^	13	15	14	14	ns, *p* = 0.912
**Age (Years)**	23.0 ± 4.6	23.1 ± 4.0	22.0 ± 3.6	22.8 ± 4.5	ns, *p* = 0.714
**Weight (kg)**	61.7 ± 1.5	69.0 ± 2.4	64.9 ± 2.5	64.1 ± 1.7	ns, *p* = 0.087
**Education Level (1–5)**^#^	2.7 ± 0.2	2.8 ± 0.1	2.6 ± 0.1	2.7 ± 0.1	ns, *p* = 0.727
**Digit Span (Forwards + Backwards)**^#^	13.1 ± 0.4	13.0 ± 0.5	12.7 ± 0.4	13.8 ± 0.5	ns, *p* = 0.252
**Impulsivity: BIS-11**	61.9 ± 1.5	65.4 ± 1.5	64.7 ± 1.6	63.3 ± 2.0	ns, *p* = 0.465
**Risk Taking: DOSPERT (Total)**	104.3 ± 3.0	113.5 ± 3.6	107.7 ± 3.7	105.5 ± 3.5	ns, *p* = 0.245
**Distractibility: CFQ**	38.3 ± 1.9	41.7 ± 1.6	43.9 ± 1.8	40.3 ± 1.9	ns, *p* = 0.185
**Sleep Quantity on the Previous Night (Hours)**^#^	7.3 ± 0.2	7.2 ± 0.2	6.7 ± 0.3	7.1 ± 0.2	ns, *p* = 0.513
**Sleep Quality on the Previous Night (1–8)**^#^	5.5 ± 0.3	5.7 ± 0.2	5.3 ± 0.3	5.4 ± 0.2	ns, *p* = 0.620
**Fatigue during Task (0–100)**	44.6 ± 3.9	44.8 ± 3.6	43.5 ± 2.6	41.2 ± 3.7	ns, *p* = 0.876
**Active Drug (%)**^#^	50	77	86	44	*p* = 0.001

Between-group comparisons revealed no significant differences (ns = nonsignificant) for gender, age, body weight, education level, baseline cognitive capacity (Digit Span), impulsivity (Barratt Impulsiveness Scale, BIS-11), risk taking (Domain-Specific Risk-Taking Scale, DOSPERT), distractibility (Cognitive Failures Questionnaire, CFQ), fatigue during the task, or sleep quality or quantity on the previous night. For continuous data, one-way ANOVAs were used to test for any between-group differences. For discrete data (^#^), Kruskal-Wallis tests were applied. Education level refers to the highest attained from the following: 1 = compulsory education (≤12 y); 2 = further education (13–14 y); 3 = undergraduate degree (15–17 y); 4 = one postgraduate degree (≥18 y); and 5 = multiple postgraduate degrees. Age data are mean ± standard deviation (SD). Remaining data are mean ± SEM. Active drug refers to the percentage of participants within each group who reported at the end of the experiment that they believed they had received an active drug. http://dx.doi.org/10.6084/m9.figshare.3168682.v1.

^#^ Discrete data.

On each trial, participants were required to respond to the presentation of one of four visual stimuli by making a speeded button press before the end of a 1,200 ms intertrial interval (ITI) (Fig 1). Participants were trained on the stimulus-response mappings, which remained consistent within an experimental session but were counterbalanced across participants. The experimental sequence of 1,200 trials was generated by a hidden probabilistic rule that switched, without explicit indication to the participant, every 50 trials. At any given time, stimulus transitions were generated by one of eight different transition matrices (TMs). Trials were drawn from each TM three times. The order of TMs was pseudorandom, with no consecutive repeats. The overall probability of each stimulus was equal across the 1,200 trials.

This created transient contexts that participants could infer from stimulus observations, allowing them to reduce their uncertainty about events before they occurred [76]. Nonetheless, the probabilistic nature of these contexts also produced unexpected stimulus outcomes, i.e., a sensory prediction error (PE). For fast and accurate responses, participants had to track three forms of uncertainty: irreducible uncertainty arising from the inherent randomness of the probabilistic transitions between consecutive stimuli, estimation uncertainty arising from their imperfect knowledge of the probabilistic relationships governing stimulus transition contingencies within contexts, and volatility uncertainty maintained by the unsignalled contextual instability.

In total, participants responded correctly on 90.3 ± 0.79% (mean ± standard error of the mean [SEM]), 88.4 ± 1.23%, 87.7 ± 1.27%, and 89.2 ± 0.91% of the trials in the Placebo, NA-, ACh-, and DA- groups, respectively. The percentages of correct responses did not differ between groups (F_3,123_ = 1.12, *p* = 0.345). Since a significant time x drug interaction on self-reported alertness was identified (see the Physiological and Subjective Control Measures results in S1 Text), the participant-specific difference in alertness between baseline and the time corresponding to peak drug concentration, *Δalertness*, was used as a covariate in our analyses to control for any interparticipant variability in subjective drug effect. Since psychopharmacological drugs can have dose-dependent effects [77–79], we also included body weight as an additional covariate in our analyses.

### Model-Agnostic Analyses

**Fig 2:**
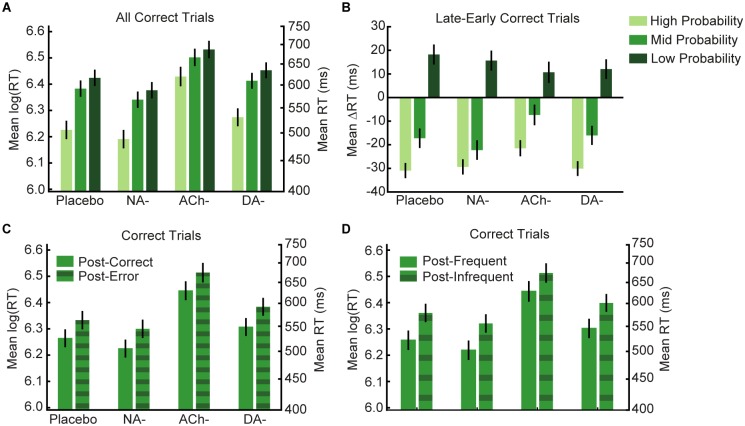
Model-agnostic changes in log(RT) indicate that participants learned to predict the stimulus transitions. (A) In all four groups, log(RT) increased as a stimulus’ true transition probability decreased. (B) A median split on each 50-trial contextual block was used to compare mean log(RTs) on Early (1–25) and Late (25–50) trials at each probability level. Over the course of a context, participants became faster at responding to High and Mid probability stimuli and slower at responding to Low probability stimuli. (C) Across drug groups, participants showed evidence of post-error slowing on correct trials that followed an erroneous response compared to those following correct responses. (D) They also showed evidence of slowing on correct trials that followed an infrequent stimulus transition. Results are mean ± SEM, corrected for the covariates Δalertness and body weight. Results shown in A, B, and D were modulated by drug group. http://dx.doi.org/10.6084/m9.figshare.3796353.v1.

We first conducted a series of conventional, model-agnostic analyses of behaviour, to assess whether participants learned about the underlying stimulus transition contingencies and whether learning was influenced by our pharmacological interventions (Fig 2). First, we conducted a repeated-measures analysis of variance (RM-ANOVA) of the log(RTs) for correct responses on trials binned according to the five true conditional probabilities that existed in each of the TMs, grouped into High (0.85 and 0.70), Mid (0.25 and 0.20), and Low (0.05) transition probabilities, with drug as a between-subject factor. This revealed a significant decrease in log(RTs) with increasing transition probability (main effect of probability: F_1.27,150.10_ = 28.32, *p* < 0.001, effect size η_p_^2^ = 0.19), which was modulated by drug type (probability x drug interaction: F_3.82,150.10_ = 12.33, *p* < 0.001, η_p_^2^ = 0.24).

Moreover, across the course of a contextual block (Fig 2B), participants became faster at responding to High and Mid probability stimuli and slower at responding to the Low probability stimuli (significant main effects of probability: F_1.28,151.41_ = 27.80, *p* < 0.001, η_p_^2^ = 0.19, and time: F_1,118_ = 12.01, *p* = 0.001, η_p_^2^ = 0.09; probability x time interaction: F_2,236_ = 6.55, *p* = 0.002, η_p_^2^ = 0.05). The effect was modulated by drug type (probability x time x drug interaction: F_6,236_ = 3.16, *p* = 0.005, η_p_^2^ = 0.07) but again not systematically related to differences in Δalertness or body weight between drug groups (all *p* > 0.05). Post hoc (Benjamini-Hochberg-corrected) pairwise comparisons indicated that the impact of drug was driven by the ACh- group, which showed significant log(RT) slowing compared to Placebo (t_57_ = 2.98, *p* = 0.003, effect size Cohen’s *d* = 0.79). Together, these results indicate that participants learned about the true stimulus transition contingencies and that this learning was modulated by our pharmacological manipulations.

As is common in such behavioural tasks [80–83], participants showed evidence of post-error slowing on correct trials following those on which they made an error (F_1,118_ = 10.92, *p* = 0.001, η_p_^2^ = 0.09; Fig 2C). This effect was not modulated by drug group (trial-type x drug interaction: *p* = 0.933) or by Δalertness or body weight (both *p* > 0.29). Participants also demonstrated significant log(RT) slowing on correct post-infrequent trials (true transition probability = 0.05) compared to all other correct trials (F_1,118_ = 36.00, *p* < 0.001, η_p_^2^ = 0.23; Fig 2D), which was modulated by drug group (F_3,118_ = 4.58, *p* = 0.005, η_p_^2^ = 0.10), but not by Δalertness or body weight (both *p* > 0.15). This effect was driven by the ACh- group, with pairwise comparisons revealing significant slowing compared to Placebo (t_57_ = 3.38, *p* = 0.001, *d* = 0.90). Error rates significantly decreased with increasing transition probability (main effect of probability: F_1.57, 182.11_ = 5.04, *p* = 0.013, η_p_^2^ = 0.04). The effect was again modulated by drug type (probability x drug interaction: F_4.71, 182.11_ = 4.72, *p* = 0.001, η_p_^2^ = 0.11), but not by Δalertness or body weight (both *p* > 0.78). There was no between-subject effect of drug group (*p* = 0.768). For additional model-agnostic analyses, please refer to S1 Text. Raw RT data can be found at https://dx.doi.org/10.6084/m9.figshare.3793410.v2.

### Model-Based Analyses

**Fig 3:**
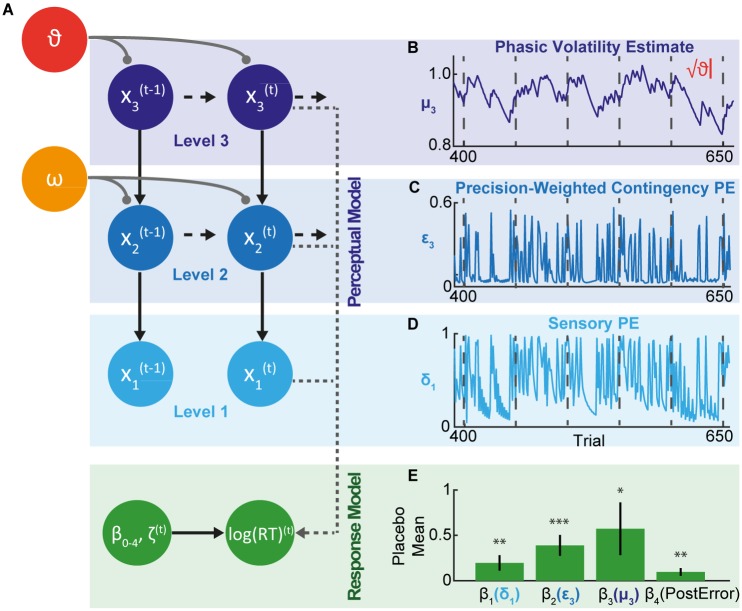
Hierarchical Gaussian Filter (HGF). (A) The perceptual model tracks an individual’s learning of the task’s structure across three levels. State ***x***_1_ represents trialwise stimulus transitions from one stimulus to the next, ***x***_2_ the probability of the transitions (i.e., transition contingencies), and *x*_3_ the phasic volatility, where *t* is the current trial number. Participants hold and update beliefs about the true quantities at each level, with a mean μ and a variance σ. ϑ and ω are participant-specific parameters that couple the levels and determine the respective speed of belief updating about phasic volatility and transition contingencies. The response model describes the mapping from a participant’s trialwise beliefs onto their observed log(RT) responses. (B) Example of the trialwise dynamics at level 3 from Placebo Participant 2. μ_3_ reflects the participant’s belief about the true phasic volatility (*x*_3_). Vertical dashed lines indicate true context switches. μ_3_ tends to increase following a context change and then decreases over the course of a context as the participant learns the new contextual rule and thus perceives the environment to be increasingly stable. √ϑ is a variance determining the step-size of μ_3_ and therefore how quickly the participant updates their phasic volatility estimates. (C) As in B, but for precision-weighted contingency prediction error [PE] (ε_3_) at level 2. This estimate results from weighting the contingency PE (δ_2_) by a precision ratio that captures uncertainty about input from the level below relative to the level above. The higher the precision at level 2, the more meaningful a deviation from the predicted stimulus transition contingency. This in turn increases the impact on phasic volatility belief updating at level 3. For simplicity, we only depict the ε_3_ trajectory for true transition changes. (D) As in B and C, but for sensory PE (δ_1_) at level 1. This estimate arises from irreducible uncertainty about stimulus transitions. Trialwise values are equivalent to $1-{\hat{x}}_{1}$, where ${\hat{x}}_{1}$ is equal to the participant’s trialwise prediction of the occurring transition. Again, for simplicity we only show δ_1_ values for true transitions here. (E) Mean β values for the Placebo group indicate that increases in sensory PE (β_1_), precision-weighted contingency PE (β_2_), and phasic volatility estimates (β_3_) slowed participants’ trialwise log(RTs). There was also evidence of post-error slowing (β_4_). Results are mean ± SEM. * *p* < 0.05, ** *p* < 0.01, *** *p* < 0.001. http://dx.doi.org/10.6084/m9.figshare.3796356.v1.

The HGF model (Fig 3) allows us to map an individual’s beliefs about stimulus transitions, transition contingencies, and volatility—and the respective irreducible, estimation, and volatility uncertainty about these beliefs—onto his/her observed RT responses. The HGF is hierarchical not only in that learning occurs simultaneously at multiple levels, but also in that belief updating at one level is constrained by beliefs at the level above. This provides a generic framework for implementing dynamic learning rates, which underlie learning in volatile environments [1,84]. We predicted that antagonising NA and ACh would impact on participants’ computation of volatility uncertainty and estimation uncertainty respectively, while DA antagonism would impede motor adaptation to uncertain outcomes without perturbing the course of learning.

#### Perceptual model

The HGF’s perceptual model tracks a participant’s learning of the task’s structure: the trialwise stimulus transitions at level 1, the probability of the transitions (i.e., transition contingencies) at level 2, and the volatility of transition contingencies at level 3 (Fig 3A). Trialwise trajectories of each participant’s perceptual estimates at each level evolve according to the predictions made and outcomes experienced by that individual (Fig 3B–3D). At levels 2 and 3, these estimates are modelled by Gaussian distributions with a mean (μ) and variance (σ), the latter reflecting the uncertainty of the estimate. Precision (π) of the estimate is equal to inverse variance (1/σ). Irreducible uncertainty at level 1 gives rise to sensory PE, **δ**_1_. Estimation uncertainty at level 2 gives rise to contingency PE, **δ**_2_. We can weight PEs according to their precision (inverse uncertainty). At level 1, this gives us precision-weighted sensory PE, **ε**_2_, and at level 2 precision-weighted contingency PE, **ε**_3_. Volatility uncertainty arises from phasic volatility beliefs, μ_3_, at level 3. See Materials and Methods and S1 Text for full details.

Importantly, the HGF does not assume fixed learning across the population but rather contains participant-specific parameters that couple the hierarchical levels and allow for individual expression of approximate Bayes-optimal learning. ϑ determines the speed of learning about volatility, i.e., the rate at which estimates of phasic volatility (μ_3_) are updated. As such, ϑ encapsulates metavolatility, i.e., the rate at which volatility changes, with higher values implying a belief in a more unstable world and leading to a more variable learning rate (as expressed in phasic volatility belief updating). By contrast, ω is a constant component of the volatility and captures how rapidly individuals generally update their beliefs about transition contingencies at level 2. Changes in ω therefore lead to a tonic alteration of the learning rate. Comparing ϑ and ω estimates for each of the drug groups to the Placebo group allowed us to assess the effects of NA, ACh, and DA antagonism on perceptual belief updating.

We note that while model-agnostic analyses can provide an indication of learning and possible drug effects, the model we endorse here permits us to probe (our best guess at) subjective expectations about the transitions that are driven by data-limited observations. Moreover, it separates the set of relatively complex and interacting factors that influence RTs in a computationally limpid way and provides us with insight into the individual effects of our pharmacological manipulations.

**Fig 4:**
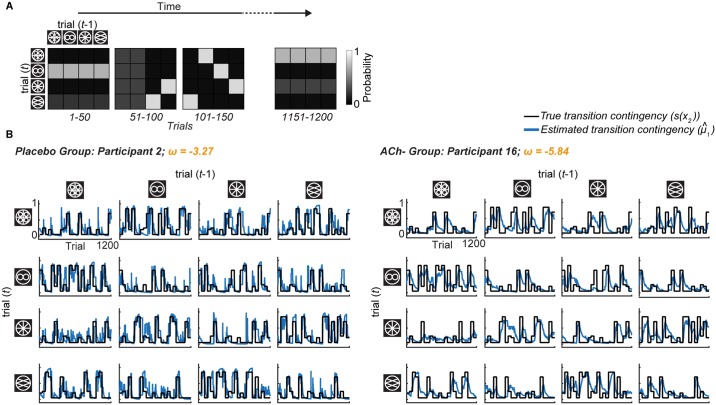
True and estimated stimulus transition contingencies for two example participants. (A) Transitions between pairs of stimuli, from trial *t-1* to trial *t*, were defined by TMs. Every 50 trials, the TM switched to a different matrix. (B) Each panel corresponds to 1 of 16 possible transitions between stimuli across 1,200 trials. The black lines indicate the true transition contingencies. The blue lines reflect the participant’s inferred estimates (i.e., the posterior expectation of these contingencies, ${\hat{\mu }}_{1}$), before seeing the stimulus outcome on each trial. The model tracked the true underlying contingencies and detected change points. In a representative participant from the Placebo group, the model tracked the true transition contingencies closely. An example participant from the ACh- group showed a greater discrepancy in the tracking of the true transition contingencies. This is reflected in the two participants’ ω estimates: Placebo Participant 2 showed a higher transition contingency learning rate (ω = −3.27) than ACh- Participant 16 (ω = −5.84). http://dx.doi.org/10.6084/m9.figshare.3796362.v2.

Overall, the model tracked the true stimulus transitions well (Fig 4). We note that the model is uninformed about the true stimulus transition probabilities but rather bases its estimates on the observed stimulus transitions only. The punctate change points contained in the true generative process are detected implicitly by the HGF as an increase in learning rate (α_1_), which reflects the influence of increased uncertainty and formally corresponds to a reduced contribution of belief precision (denominator in Eq 3) to the weighting of PE.

**Fig 5:**
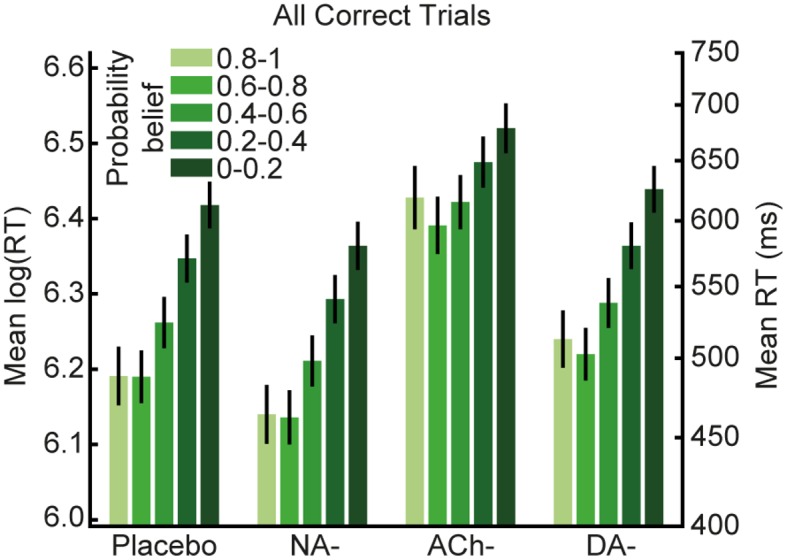
Model-based changes in log(RT) mirror our model-agnostic finding that participants learned to predict the stimulus transitions. In all four groups, faster responses were observed as participants’ estimates of the true transition contingencies increased, mirroring our model-agnostic result (see Fig 2A). Results are mean ± SEM, corrected for Δalertness and body weight. http://dx.doi.org/10.6084/m9.figshare.3796401.v2.

Moreover, when we categorise trials according to participants’ trialwise estimates of transition contingencies, as provided by model parameter ${\hat{\mu }}_{1}$ (five bins: 0.8–1, 0.6–0.8, 0.4–0.6, 0.2–0.4, and 0–0.2), we observe the same decrease in log(RT) with increasing transition probability established in our model-agnostic results (cf. Fig 5 with Fig 2A; significant effect of ${\hat{\mu }}_{1}$: F_1.67,185.64_ = 17.56, *p* < 0.001, η_p_^2^ = 0.14). As in the model-agnostic results, this was modulated by drug group (significant ${\hat{\mu }}_{1}$ x drug interaction: F_5.02,185.64_ = 9.51, *p* < 0.001, η_p_^2^ = 0.20), but not by Δalertness or body weight (both *p* > 0.11).

#### Response model

The response model describes the mapping from a participant’s perceptual trialwise beliefs, as provided by the perceptual model, onto their observed log(RT) responses (Fig 3A). The response model we report in the following (Eq 1) describes log(RT) as a linear function of a constant component of log(RT), sensory PE (δ_1_), precision-weighted contingency PE (ε_3_), phasic volatility (μ_3_), post-error slowing, and Gaussian noise (ζ). (See Materials and Methods and S1 Text for random effects Bayesian model comparison of alternative response models.)

$$\text{log}{\left(\text{RT}\right)}^{\left(\text{t}\right)}={\;\beta }_{0}+{\;\beta }_{1}(\delta_{1}^{\left(\text{t}\right)})\;+{\;\beta }_{2}(\varepsilon_{3}^{\left(\text{t}\right)})\;+{\;\beta }_{3}(\mu_{3}^{\left(\text{t}\right)})\;+{\;\beta }_{4}({\text{PostError}}^{\left(\text{t}\right)})\;+{\;\zeta }^{\left(\text{t}\right)}$$ (1)

**Fig 6:**
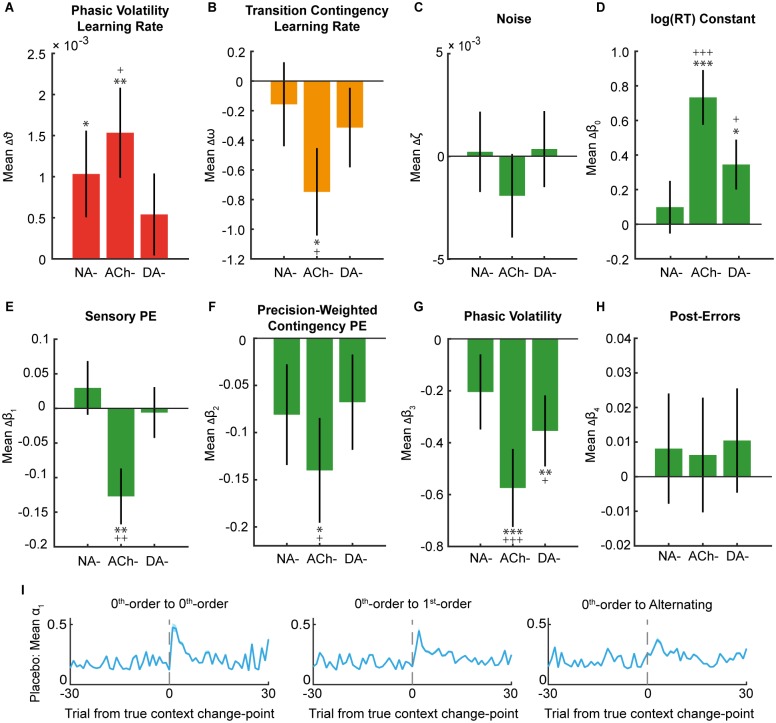
Model parameter results. (A–H) Compared to the Placebo group, NA and ACh antagonism modulated participants’ perceptual belief updating. NA- increased the rate at which participants updated their volatility estimates (increased ϑ). ACh- decreased the rate at which participants learned about stimulus contingencies (decreased ω) and increased participants’ ϑ estimates. DA- antagonism decreased the sensitivity of participants’ trialwise responses to their phasic volatility estimates (β_3_). DA- and ACh- antagonism also caused some general response slowing (β_0_). The three drug groups and the Placebo group showed equivalent post-error slowing (β_4_) and Gaussian noise (ζ). Results are (mean Drug) − (mean Placebo), ± the standard error of the difference (SED) between the means of the two samples and corrected for the covariates Δalertness and body weight. For uncorrected comparisons: * *p* < 0.05, ** *p* < 0.01, *** *p* < 0.001. For corrections for three comparisons, ^+^’s are used for Benjamini-Hochberg-corrected significance thresholds at ^+^ *p* < 0.05, ^++^ *p* < 0.01, ^+++^ *p* < 0.001. See S2 Table for Placebo group means. (I) First-level learning rate (α_1_) trajectories for truly occurring trials from the Placebo group. Increases in α_1_ are observed following a true change in context. This α_1_ increase is amplified for a more obvious switch from one easy-to-detect zeroth-order context to a different zeroth-order context. In contrast, a switch to an alternating context, which is trickier to detect, is accompanied by a modest, more gradual increase in α_1_. Results are mean ± SEM. http://dx.doi.org/10.6084/m9.figshare.3796407.v1.

All regression coefficients for the Placebo group were significantly greater than 0 (Fig 3E), meaning that sensory PE (β_1_(δ_1_): t_29_ = 2.63, *p* = 0.010, *d =* 1.38), precision-weighted contingency PE (β_2_(ε_3_): t_29_ = 3.74, *p* < 0.001, *d =* 1.97), and phasic volatility estimates (β_3_(μ_3_): t_29_ = 2.05, *p* = 0.043, *d =* 1.08) all had slowing influences on log(RT) and that there was evidence of post-error slowing (β_4_(PostError): t_29_ = 3.09, *p* = 0.003, *d =* 1.62). Each of the drug groups showed equivalent post-error slowing to the Placebo group (all *p* > 0.48; Fig 6H), mirroring our model-agnostic result. The lack of a difference in the noise parameter ζ between the Placebo group and any of the drug groups (all *p* > 0.34; Fig 6C) indicates that the model’s ability to predict log(RT) was unaltered under our drug manipulations. Comparing the regression coefficients for each drug group to the Placebo group allowed us to assess whether NA, ACh, and DA antagonism altered the mapping from perceptual beliefs onto motor responses.

### The Influence of NA and ACh in Perceptual Uncertainty Computations

#### NA antagonism increased phasic volatility learning rate

The noradrenergic (α1-receptor) antagonist prazosin increased the rate at which individuals updated their volatility estimates, as reflected by an increase in ϑ (linear model: t_59_ = 2.11, *p* = 0.037, effect size Cohen’s *d =* 0.56; Fig 6A). A higher ϑ leads to greater fluctuations in participants’ phasic volatility estimates, μ_3_, resulting in a more variable phasic learning rate. By contrast, there was no effect on ω (*p* = 0.574; Fig 6B), indicating that the tonic learning rate about the probabilistic contexts remained unchanged. We note that when we apply a Benjamini-Hochberg correction for three comparisons, the reported effect of NA- on ϑ does not survive. However, we do observe a significant increase in ϑ for the NA- group when we run permutation tests randomising over drug assignment (see S1 Text and S5 Table for details). Further, when we rerun the linear model analyses excluding body weight, which was equivalent across drug groups (F_3,120_ = 2.24, *p* = 0.087), as a covariate, the significant increase in ϑ for the NA- group survives the more stringent correction for three comparisons (t_60_ = 2.32, *p* = 0.022, *d* = 0.60), and all other reported significant effects are unchanged.

#### ACh antagonism slowed learning about stimulus transition contingencies

Muscarinic ACh receptor antagonism under biperiden had more widespread perceptual effects. While ϑ was again significantly increased compared to Placebo (t_57_ = 2.86, *p* = 0.005, *d =* 0.79), ω estimates in the ACh- group were significantly reduced (t_57_ = −2.59, p = 0.011, *d = −*0.71). The lower estimate of ω indicates that participants were slower to update their transition contingency estimates under biperiden and thus slower to adapt to the probabilistic contexts.

#### DA antagonism had no effect on learning about task structure

The D1/D2 DA receptor antagonist haloperidol did not influence the rate at which participants learned about the task’s volatility or contextual transition contingencies compared to Placebo (ϑ and ω: both *p* > 0.23).

To summarise, both NA and ACh antagonism altered learning of uncertain events arising from unexpected contextual changes in the environment. Only ACh antagonism disrupted learning of transition contingencies within probabilistic contexts.

### Neuromodulatory Effects on Response Modulation

#### NA antagonism had no influence on responses

The response model output revealed no significant effects of NA antagonism on participants’ capacity to modulate their motor responses according to their perceptual estimates of uncertainty (Fig 6E–6G).

#### ACh antagonism reduced response sensitivity to perceptual beliefs

Compared to Placebo, ACh antagonism reduced the sensitivity of participants’ motor responses to sensory PE (β_1_: t_57_ = −3.25, p = 0.002, *d = −*0.90), precision-weighted contingency PE (β_2_: t_57_ = −2.56, p = 0.012, *d = −*0.71), and phasic volatility estimates (β_3_: t_57_ = −3.91, p < 0.001, *d = −*1.08) (Fig 6E–6G).

#### DA antagonism reduced response sensitivity to phasic volatility

Compared to Placebo, DA antagonism led to a decrease in the influence of phasic volatility estimates on log(RT) (β_3_: t_60_ = −2.65, p = 0.009, *d = −*0.67; Fig 6G). This indicates that DA antagonism suppressed the sensitivity of motor responses to higher-level inference.

In addition to the effects reported above, the log(RT) constant output indicated that suppressing DA and ACh also led to some general log(RT) slowing (β_0_: t_60_ = 2.48, *p* = 0.015, *d =* 0.62; t_57_ = 4.78, *p* < 0.001, *d =* 1.32, respectively; Fig 6D). Subjective Δalertness systematically modulated the effects observed on ϑ (t_118_ = 2.62, *p* = 0.010, *d* = 0.02), sensory PE (β_1_: t_118_ = 2.52, *p* = 0.013, *d* = 0.02) and precision-weighted contingency PE (β_2_: t_118_ = −3.21, p = 0.002, *d* = −0.02). None of the effects were systematically modulated by any between-group differences in body weight (all *p* > 0.10).

### Control Analyses

In S1 Text, we provide details of the physiological and subjective control measures. We also provide additional assessments of the HGF model and its ability to provide a good fit to the behavioural data by examining model parameter correlations, residuals and simulations (S2–S6 Figs). Finally, we report the results of additional permutation tests (S5 Table), which allow us to make distribution-free comparisons of the effects of our drug manipulations on the model parameters.

## Discussion

By implementing a novel probabilistic serial RT task in conjunction with three pharmacological manipulations and placebo, we characterised the roles of three neuromodulatory systems during perceptual belief updating and response selection. Leveraging a hierarchical Bayesian learning model to decompose hierarchically related forms of uncertainty enabled us to pinpoint processes linked to NA, ACh, and DA. While manipulating NA and ACh modulated perceptual uncertainty computations, DA receptor antagonism reduced the sensitivity of the motor system to uncertainty estimates.

A key benefit of our pharmacological approach is that we were able to directly manipulate the function of three different neuromodulatory systems and compare the resulting psychopharmacological effects to a placebo condition. This is relevant given likely functional overlap between the different neuromodulatory systems, as observed here. Indeed, manipulation of a single neuromodulatory system, or use of a single drug, will be agnostic to such an overlap and may make any one effect appear more relevant and specific than it is. We are also able to extend interpretations of earlier neuroimaging studies [15,16], from which it is not possible to infer with certainty that activations in particular brain regions, with inhomogeneous cellular compositions, reflect the activity of specific neuromodulatory neurons.

### Overlapping, but Dissociable, Noradrenergic and Cholinergic Influences on Perceptual Belief Updating

We found considerable overlap in the influence of NA and ACh antagonism on perceptual belief updating but also quantitative differences between drug conditions. While part-synergistic, part-antagonistic interactions between the two neuromodulators during uncertainty processing have been theorised previously [4], to our knowledge this is the first study to directly assess these putative computational roles, and to distinguish them from dopaminergic effects, under three pharmacological manipulations and within the same computational framework. We propose that ACh guides probabilistic learning within environmental contexts, while NA has a more circumscribed role in modulating the rate at which an agent learns about the volatility latent in the environment.

#### NA influences beliefs about unexpected environmental changes

Our results suggest that NA antagonism under prazosin altered the rate at which individuals updated their volatility beliefs, as indicated by an increase in the model parameter ϑ. We note, however, that this result should be interpreted conservatively since it did not survive correction for three comparisons. Nonetheless, a permutation test, without any distributional assumptions, mirrored the result of the uncorrected comparisons (see S5 Table). Moreover, the result did survive correction when body weight (which was equivalent across drug groups) was excluded as a covariate from the linear model analyses. An influence of NA- on ϑ fits with the theorised role for NA in computing uncertainty arising from changes in environmental context [4]. Numerous studies have offered evidence that the NA system is sensitive to highly unexpected events that arise from a hidden contextual change. Noradrenergic neurons in the rat and nonhuman primate LC are responsive to environmental novelty and unexpected changes in reward contingencies [48–50,85]. Additionally, changes in pupil diameter, attributed at least in part to noradrenergic LC activity [53,68,86], have been shown to correlate with unexpected outcomes [54,55].

More specifically, in the present study we observed faster volatility belief updating following NA antagonism. In our model, ϑ represents the volatility of the volatility, and thus, our results suggest that NA stabilises an agent’s estimate of environmental volatility. This is compatible with the notion that the volatility estimate has a relatively low baseline level, to which it returns after being pushed away. In a volatile environment, this is not an adaptive feature. Rather, the volatility estimate should remain high to enable revision of one’s beliefs. It is possible that NA prevents the volatility estimate from falling by reducing an agent’s ϑ estimate.

The neurophysiological literature has distinguished two functional modes of LC noradrenergic release [44,87]. A phasic mode, characterised by a relatively low baseline firing rate and high phasic responsiveness to task relevant stimuli, has been linked to enhanced task engagement, and a tonic mode to increased distractibility, attention shifting, and exploratory behaviour [87–89] (but see Jepma et al. [90]). More recently, human LC BOLD activity was demonstrated to correlate with “unexpected uncertainty” induced by a switch in reward probabilities associated with familiar stimuli [16], although the negative sign of this correlation still seems to lack explanation. In both our task and that used by Payzan-LeNestour et al., contextual switches required participants to identify discrete changes in underlying transitions between familiar stimuli. To continue making accurate predictions in light of new transition probabilities, participants had to increase their attentional engagement to facilitate an augmented learning rate. It is likely that in both cases a phasic LC activity mode was recruited and that this would be recognised as a decrease in BOLD activity at a neural population level. Speculatively, it also suggests that our pharmacological NA manipulation may have enabled more phasic NA responsiveness to emerge under suppression of tonic NA firing. Future investigations of the impact of noradrenergic drugs on LC activity profiles are needed to validate this theory.

#### ACh balances the attribution of uncertainty within and between environmental contexts

Muscarinic ACh receptor antagonism by biperiden led to slower updating of beliefs about stimulus transition contingencies, and therefore slower adaptation to the probabilistic contexts, as reflected by a decrease in the model parameter ω. We argue that this slowed adaptation also had knock-on effects higher up in the inferential hierarchy. Namely, we propose participants attributed perceived violations of their expectations to gross contextual switches as opposed to chance fluctuations in stimulus outcomes, which would be expressed as an increase in ϑ. In light of previous work, which we discuss next, it seems reasonable to suggest that by setting the rate at which an agent learns probabilistic associations, ACh facilitates the appropriate attribution of violated expectations to chance fluctuations in an environment’s statistical regularities or to gross switches in environmental context.

According to the structure of our Bayesian model, a reduction in ω maps onto a reduced precision weighting of perceptual belief updates at level 2; compare Eq 2 and Equation B in S1 Text. Our findings indicate that under biperiden less weight was given to sensory evidence, and updates of probability estimates became more reliant on current beliefs. This supports proposed roles for ACh in regulating the relative influences of stimulus-driven versus expectation-guided processing [52,91] and attentional deployment [37,40]. For instance, it has been shown that pharmacologically stimulating ACh augments bottom-up sensory signalling in human primary auditory cortex in response to auditory stimuli, possibly by enhancing the gain of superficial pyramidal cells, to bias inference towards sensory data [30].

In a recent study, Vossel et al. examined perceptual belief updating during a probabilistic attentional cueing paradigm. By applying a similar instantiation of the HGF to saccadic RTs, the authors demonstrated faster learning about contextual probabilities following administration of galantamine, an acetylcholinesterase inhibitor which increases the synaptic availability of ACh, as indicated by an increase in model parameter ω [17]. In the present study, we observe the opposite behavioural effect with the opposite pharmacological manipulation (ACh receptor antagonism), offering independent evidence that ACh signalling guides belief updating about probabilistic associations within environmental contexts.

Our results also indicate that ACh antagonism led individuals to update their volatility estimates more rapidly, reflected by an increase in the model parameter ϑ. This is consistent with the notion that ACh- participants’ impaired ability to learn transition contingencies led them to infer that contexts changed at a faster rate. Notably, in their theoretical framework, Yu and Dayan predicted that ACh depletions should cause an agent to underestimate the amount of randomness in a given context. In turn, this causes chance events occurring within a context to seem more significant than they are, and thus, they are more likely to be incorrectly taken as indicative of a context change (see Fig 6D in Yu and Dayan 2005 [4]). Our experimental observations support this hypothesis and are compatible with data indicating that cholinergic antagonists increase distractibility [92], while agonists suppress it [93–95].

We note that, although the quantities used in this current work are not identical to those previously introduced by Yu and Dayan 2005 [4], the HGF does embody versions of the same forms of uncertainty. The highest level of uncertainty in Yu and Dayan’s (YD’s) framework was induced by abrupt, discrete changes in contingencies, which induced what YD call “unexpected uncertainty” (and ascribed to NA). By contrast, the highest level of uncertainty in the HGF is the overall instability of the world, i.e., the rate at which volatility changes. It is this that we found to be modulated by our NA antagonist. Conversely, YD’s notion of “expected uncertainty” (ascribed to ACh) suggests that it arises from the known unreliability of predictive relationships within a familiar environmental context. Amongst other effects, the lower the expected uncertainty, the slower the learning—consistent with the effect of parameter ω in the HGF, which was found to decrease under cholinergic antagonism. Along with YD, we also argue that this change in learning has further knock-on effects for what participants perceive to be a chance random event or a change of context (and hence unexpected uncertainty).

In sum, our findings offer empirical support for the theoretical proposal that ACh and NA interact to construct appropriate cortical representations of volatile contexts, which facilitates optimal inferences about the current environment [4]. By regulating high-level uncertainty representations, the two neuromodulators contribute to the updating of one’s perceptual beliefs, both within and between environmental contexts, an idea that is broadly supported by recent neuroimaging [15,16] and pharmacological [17] evidence.

### DA Sensitises Motor Responses to Environmental Volatility

As per its construction, our computational model allowed us not only to characterise perceptual belief updating under our three pharmacological manipulations but also to assess how each intervention influenced the deployment of motor responses in response to individual estimates of uncertainty. Pharmacologically manipulating DA and ACh altered the degree to which participants’ perceptual beliefs modulated the preparation of their speeded responses to uncertain stimuli. In contrast, NA antagonism had no significant impact on the sensitivity of participants’ motor responses to their current perceptual beliefs relative to placebo.

We had originally predicted that an individual’s capacity to modulate response selection following a sensory PE would be dependent on DA. Indeed, it has previously been shown that pharmacological DA depletion impedes adaptive reactions to unexpected events occurring within predictable contexts [58]. However, in the present study, we found no evidence that DA receptor antagonism influenced participants’ reactions to low-level sensory PE (δ_1_). Rather, we observed that suppressing DA significantly reduced β_3_, which we interpret as a reduction in the sensitivity of participants’ motor responses to their higher-level volatility estimates (μ_3_).

It is important to note that some key differences distinguish our present experimental design from previous paradigms. In earlier work, participants were pre-trained to respond to stimuli presented within one predictable context, defined by one TM. Furthermore, switches from predictable to unpredictable contexts, consisting of random presentations of stimuli, were explicitly signalled [58]. Therefore, any probabilistic learning and higher-level perceptual uncertainty were removed. In this earlier setting, dopaminergic antagonism under haloperidol selectively impaired participants’ reactions to unexpected events that elicited large sensory PEs.

In contrast, our present task created a more complex, and arguably more ecologically valid, scenario in which individuals had to infer the current context for themselves and adapt to any contextual changes. Here, uncertainty representations had to be acquired through direct sampling from a distribution of observations. To our knowledge, our study is the first attempt to interrogate the impact of DA, ACh, and NA on nonrewarded probabilistic learning within a single behavioural paradigm and a unified Bayesian framework. By estimating beliefs about various forms of uncertainty, we sought to identify neuromodulatory contributions specifically related to particular forms of uncertainty, as opposed to any confounding variables.

Our finding that haloperidol reduced the sensitivity of participants’ responses to their phasic volatility estimates does sit well with an alternative line of work highlighting the importance of DA in cognitive switching [61,62]. For instance, Parkinson’s disease patients with DA dysfunction have an impaired capacity to switch from naming digits to letters when both types of stimuli are presented simultaneously, even when the task shift is explicitly cued [60]. In summary, we propose that our DA manipulation suppressed response modulation by impeding cognitive switching following complex contextual rule changes.

Muscarinic ACh receptor antagonism under biperiden also led to decreased response modulation by parameters at all three hierarchical levels: sensory PE (δ_1_), precision-weighted contingency PE (ε_3_), and phasic volatility estimates (μ_3_) compared to Placebo. We propose that ACh receptor antagonism impeded participants’ abilities to learn the statistical structure of the behavioural task, which in turn impaired their capacities to respond accordingly. Although both ACh and DA had effects on response modulation, in light of previous work, we suggest that DA’s role is to modulate motor responses according to the widespread perceptual effects of ACh.

### Limitations and Future Work

One of the main constraints of our study is that although prazosin, biperiden, and haloperidol are rather selective for NA, ACh, and DA receptors, respectively, there are complex interactions and dependencies between noradrenergic, cholinergic, and dopaminergic systems. Such interactions are a main reason why direct quantitative comparison between drug groups would not have provided direct comparisons between the action of different neuromodulators and therefore why our study was designed to detect changes relative to placebo instead. While our results highlight qualitative differences in how NA, ACh, and DA influence perceptual belief updating, future work will have to conduct direct quantitative comparisons of their roles.

Further, it is the receptors rather than the neuromodulators themselves that bring about psychophysiological effects, and there are dissociable roles of different receptor subtypes. For instance, the functions of nicotinic versus muscarinic cholinergic receptors in uncertainty signalling have yet to be directly compared. Distinctions have also been made between D1 and D2 dopaminergic receptor subtypes in regulating adaptive responses to unexpected stimuli [58]. Thus, future work could usefully be extended with genetic profiling and a range of selective agonists and antagonists for different receptor subtypes.

Finally, it is likely that all the neuromodulators operate over multiple timescales. For instance, separate, even competing, tonic and phasic effects have been a special target of investigation for NA. Teasing these timescales apart more fully is an ambition for the future, requiring a temporally richer design.

Nevertheless, our findings emphasise the necessity of studying the systems conjointly, as tasks associated with uncertainty will tend to involve them all.

### Conclusion

In summary, our results offer novel and direct insight into the complex and intricate effects of NA, ACh, and DA during a probabilistic serial RT task. Employing a hierarchical Bayesian learning model that allowed us to assess various forms of uncertainty and PEs, we provide interventional evidence linking ACh and NA to uncertainty computations within and between behavioural contexts. In contrast, DA appears to be involved in sensitising motor responses to perceptual volatility estimates. While pharmacological manipulations do not selectively target particular neuromodulatory systems, our results offer a fresh perspective on the effects of noradrenergic, cholinergic, and dopaminergic neurotransmission on the computational mechanics of perceptual belief updating according to Bayesian principles. Future studies will verify the generality of our observed effects to different behavioural paradigms with and without learning, reward, prediction, and action. By characterising uncertainty computations and response modulation, our methodology can also be used to offer fresh insight into the numerous neurological and psychiatric disorders in which there is dysregulation of processes dependent on NA, ACh, and DA.

## Materials and Methods

### Participants

A total of 128 healthy participants (56 male, aged 18–38 y, 119 right-handed) with normal or corrected-to-normal vision undertook this study. Participants gave written informed consent in accordance with the Declaration of Helsinki. The experimental protocol was approved by the UCL Research Ethics Committee (Project ID: 3491/001). The following exclusion criteria applied: history of neurological or psychiatric disease, intake of medication (other than contraceptives), smoking, recreational drug use, and current participation in other pharmacological studies. Following a screening interview to rule out intolerances or contraindications, the study clinician (DR) assigned participants pseudorandomly (i.e., ensuring a balanced distribution of gender, age, and body weight) to receive a NA, ACh, or DA antagonist or a placebo. All other authors, including the experimenter (LM), were blind to the drug conditions.

### General Procedure

We employed a double-blind, between-subject design. Each participant attended one experimental session during which they received a single oral dose of one of the following: 1 mg prazosin (α1-receptor antagonist; NA- group), 6 mg biperiden (M1-receptor antagonist; ACh- group), 2.5 mg haloperidol (D1/D2-receptor antagonist; DA- group), or a placebo. We selected doses that were in line with previous studies showing clear behavioural and neurophysiological effects [58,96–98]. On arrival, participants completed computerised versions of the Digit Span test, Barratt Impulsiveness Scale (BIS-11) [99], Doman-Specific Risk-Taking (DOSPERT) Scale [100], and Cognitive Failures Questionnaire (CFQ) [101]. Participants also self-reported their baseline mood (alertness, calmness, and contentedness) with visual analogue scales (VASs) [102], and we measured their baseline heart rate (HR) and blood pressure (BP). To assess any subjective and/or physiological drug effects, the VAS, HR, and BP measurements were repeated before participants started the RT task and again once they completed it.

Two different drug administration times were used to match peak plasma concentration across drugs, based on previous pharmacokinetic data. To ensure that participants undertook the RT task when the drug was at its most active, haloperidol was administered 2 h in advance, while prazosin and biperiden were administered 1.5 h before the main experimental session [96–98]. A random 50% of participants from the Placebo group were administered a placebo tablet at the first time point, and the other 50% at the second time point. The study clinician administered the drug or placebo while the experimenter was away from the testing room. Participants were asked not to eat for at least 1 h before the first drug administration time.

### Probabilistic Serial RT Task

Participants sat facing a computer screen positioned approximately 60 cm away. They were instructed to rest their left and right index and middle fingers on the four buttons of a custom-made button box placed in front of them and to maintain this position throughout the task. On each trial, participants were required to respond to the presentation of one of four visual stimuli by pressing an appropriate button as quickly as possible. Each stimulus was associated with one particular button. The stimulus-response mappings remained consistent within an experimental session but were counterbalanced across participants. Each participant acquired the stimulus-response mappings for their session during a training block in which they received visual error feedback after each trial. The training session comprised at least 100 trials and did not finish until the participant had reached a minimum performance criterion of 85% accuracy on the last 20 trials. Participants were then given 40 practice trials, in which the stimuli were presented in a random order and without error feedback, to familiarise them with the timings of the main experiment. An additional refresher block, consisting of at least 26 trials with error feedback, was completed immediately before the main experiment. Again, participants had to achieve 85% accuracy in the last 20 trials to proceed. On average, participants reached this criterion in 28.1 ± 1.1 trials, indicating adequate learning and retention of the mappings. There was no difference in the number of refresher trials required between groups (F_3,120_ = 1.17, *p* = 0.324).

Each participant performed 1,200 trials of the probabilistic RT task. Fig 1A shows an example trial sequence. Anticipatory responses (<80 ms RT) were recorded as incorrect. At any given time, the trial sequence was generated by one of eight TMs, which changed every 50 trials. In each case, there were 16 combinations that determined the probabilistic relationship between the stimuli presented on the current trial, *t*, and the previous trial, *t-1*. Three types of TM were utilised: first-order sequences, alternating sequences, and zeroth-order sequences (see Fig 1B and S1 Fig for further details). The order of TMs was pseudorandom, with no consecutive repeats. Importantly, the overall probability of each stimulus was equal across the 1,200 trials.

The pseudorandom order of TMs was used to generate one stimulus sequence that was used for all participants to ensure comparable learning processes and model parameter estimates. Rest periods occurred every 185 trials, orthogonal to TM switches. The importance of fast responses was stressed. Participants were told that by paying attention to any patterns in the order in which stimuli were presented and to any switches in these patterns, it might be possible to respond faster. No further information about the nature of the experiment was provided.

Combining our behavioural paradigm with three pharmacological manipulations allowed us to assess directly any separable roles for NA, ACh, and DA in belief updating under irreducible uncertainty, estimation uncertainty and volatility uncertainty and in sensitising the motor system to participants’ individual perceptual beliefs. At the end of the experimental session, participants were debriefed, indicated whether they thought they had taken an active drug or placebo, and reported the quality and quantity of their sleep on the previous night [103].

### Model-Agnostic Analyses

Trialwise RT was calculated as the time between stimulus onset and the subsequent button press. The RT data were log transformed [58]. To assess the interaction between stimulus transition probability and drug, trials were binned according to three probability levels corresponding to the presented stimuli’s true conditional probabilities as existed in the TMs (High: 0.85 and 0.70; Mid: 0.25 and 0.20; Low: 0.05) [58,59]. Mean log(RTs) for each participant’s correct responses were compared across these three probability levels and between drug groups. To obtain a model-agnostic indication of learning across the course of the probabilistic contexts, we performed a median split on each 50-trial contextual block and compared correct mean log(RTs) on Early (1–25) and Late (26–50) trials at each probability level and between drug groups. In reporting statistical differences, a significance threshold of α = 0.05 was used. Where assumptions of sphericity were violated (Mauchly’s test *p* < 0.05), the Greenhouse-Geisser correction was applied. For comparisons across the four groups, partial eta-squared (η_p_^2^) is reported as the effect size. For planned pairwise comparisons between each drug group and Placebo, we applied a Benjamini-Hochberg correction for three comparisons to the significance threshold. For pairwise comparisons, Cohen’s *d* is reported as the effect size.

### Model-Based Analyses

Model-based analyses permit quantification of the inferences participants made during the task and decomposition of the impact of different forms of uncertainty. Hierarchical Bayesian models have proven powerful for explaining the adaptation of behaviour to probabilistic contexts in volatile environments [1,15,17,70,72–74,84,104,105]. Here we developed a novel instantiation of the HGF model [9] with a focus on TMs and two components: a three-level perceptual model and a response model (Fig 3A). We implemented the model using the HGF Toolbox (http://www.translationalneuromodeling.org/tapas/). Matlab (Mathworks, United States) code can be downloaded directly from http://www.tnu.ethz.ch/de/software/tapas/download.html. Note that, for the present study, we used the tapas_logrt_linear_whatworld family of scripts. The HGF is unconstrained by normative optimality assumptions of “ideal Bayesian” models that enshrine the true characterisation of the task (or alternatively Bayesian hierarchical uncertainty about this true characterisation) [76,106,107]; instead, it allows for interindividual variability in learning by fitting parameters that determine a participant-specific approximation to Bayes optimality.

#### Perceptual model

The perceptual model is a variant of the HGF as introduced in Mathys et al. [9,10]. It tracks a participant’s learning of the task’s structure in a similar way to previous studies using the HGF [15,17,70–74]. The HGF is a generative model of observed data that provides a mapping from hidden states of the world(*x*) to sensory inputs (*u*). Unlike previous applications of the HGF, in this application the data are observed transitions between stimuli that arise from a sequence of environmental states (***x***_1_), where we use bold font to indicate a matrix. In our experiment, the *jk*^th^ element of ***x***_1_ is the transition from stimulus *k* to stimulus *j*, the probability of which participants must learn to perform the task well. There are 16 possible transitions induced by the trialwise presentation of one of four visual stimuli, meaning that ***x***_1_ is a four-by-four matrix. On each trial, an agent observes a sample from one column of the TM. Therefore, the current transition in the corresponding column of ***x***_1_ is 1, with all other elements in that column equal to 0.

The HGF comprises two parts: a generative (or forward) model that describes how data are generated, including the effect of such facets as the changing contexts, and a recognition model that performs (approximate) statistical inference on the observations of the actual data in order to determine the distribution over the values of random variables in the generative model appropriate to those particular observations. In contradistinction to models that assume that participants fashion the generative process to the task at hand, the HGF offers an inclusive scheme for explaining behaviour that generalises to a multitude of situations requiring hierarchical inference about the state of the world.

In the present application of the HGF, the generative model has two further levels above ***x***_1_. Level 2 is a four-by-four matrix ***x***_2_ of real numbers governing the transition contingencies. These undergo random walks with increments that are independent of each other. At level 3, *x*_3_ sets the variance of those random walks and therefore the rate of change (or volatility) of the elements of ***x***_2_. Since we assume that all the elements experience the same volatility (see Mathys et al. 2011; 2014), *x*_3_ is a scalar. Collectively, ***x***_2_ and *x*_3_ capture stimulus transitions and their changes over time (albeit represented heuristically as a continuous random walk in logit space with a bijective mapping to the probability of specific discrete changes). More specifically, a sample of ***x***_1_ is generated by applying a logistic sigmoid transformation to the column of ***x***_2_ associated with the stimulus that was previously shown, to generate a probability distribution over the four possible next stimuli (see S1 Text for details). A sample is then drawn from that distribution.

The recognition part of the HGF takes observations of ***x***_1_ and infers approximate posterior distributions over the values of ***x***_2_ and *x*_3_. This amounts to a variant of predictive coding in which beliefs are dynamically updated across the levels via PEs that are weighted by their salience, or expected precision (equivalent to inverse variance, or uncertainty). Estimates of stimulus transition probabilities, i.e., the posterior distribution over ***x***_2_, correspond to the posterior distribution over ***x***_2_ and are updated by PEs about stimulus occurrences. Estimates of environmental volatility, i.e., the posterior distribution over *x*_3_, are updated in proportion to PEs about the transition contingencies. Thus, the effective learning rate is influenced by uncertainty about current beliefs and environmental stability.

More precisely, trial by trial, participants update their beliefs about the true quantities at each level, which at levels 2 and 3 are modelled by Gaussian distributions with a mean (μ) and variance (σ), the latter reflecting the uncertainty of the estimate. Precision ($\hat{\pi }$) of the prediction is equal to the inverse variance ($\text{1/}\hat{\sigma }$), where the hat denotes the participant’s predicted estimate before seeing the stimulus outcome on each trial. At level 1, when the elements of ***x***_2_ are each transformed by the logistic sigmoid to produce probabilities ${\hat{x}}_{1}$ there is irreducible uncertainty (the participant’s estimate of which is written as ${\hat{\mu }}_{1}$), which gets its name since it is undiminished by learning [11]. Irreducible uncertainty arises from any probabilistic relationship and is closely related to entropy, with an inverted-U relationship to probability that peaks at *p* = 0.5. The quantity gives rise to sensory PE *(****δ***_*1*_*)* following the presentation of an unexpected stimulus that would require a participant to respond against their expectation.

Note that we do not enforce columnwise normalisation of ${\hat{x}}_{1}$ (i.e., the columns do not necessarily add up to one, as they would have to in order to represent a proper probability distribution over mutually exclusive events). We argue that ensuring that the probabilities sum to one would require a sort of certainty about the stimuli that participants do not necessarily have when performing the task; for instance, it would require precise a priori knowledge that each and every trial will present exactly one of four stimuli and that there is no possibility of novel stimuli occurring during the experiment. In practice, the statistics governing the sensory events that occurred in our task ensure that column sums of participants’ ${\hat{\mu }}_{1}$ estimates never stray far from unity.

At level 2, ${\hat{\sigma }}_{2}$, which is informational in origin, represents estimation uncertainty about the true probabilistic relationships governing stimulus transitions, giving rise to a more abstract contingency PE (**δ**_2_). At level 3, volatility uncertainty arises from the environment’s volatility, i.e., how quickly the transition contingencies are changing.

Generally, at any level *i* of the hierarchy, the update of the belief on trial *t* (i.e., posterior mean $\mu_{i}^{\left(t\right)}$ of the state *x*_*i*_) is proportional to the precision-weighted PE, $\varepsilon_{i}^{\left(t\right)}$. This weighted PE is the product of the upward-propagating PE, $\delta_{i-1}^{\left(t\right)}$, and a precision ratio, $\psi_{i}^{\left(t\right)}$(see S1 Text for details), capturing the uncertainty about input from the level below relative to the uncertainty about the state of the level being updated [15]. A general and didactically useful form of this precision-weighted PE (with subtle differences below level 3; see Mathys et al. 2014 [10]) is:

$$\Delta \mu_{i}^{\left(t\right)}\propto \varepsilon_{i}^{\left(t\right)}=\psi_{i}^{\left(t\right)}\;\delta_{i-1}^{\left(t\right)},$$ (2)

$$\psi_{i}^{\left(t\right)}\;=\frac{{\hat{\pi }}_{i-1}^{\left(t\right)}}{\pi_{i}^{\left(t\right)}}\;.$$ (3)

Thus, precision-weighted sensory PE (**ε**_***2***_) is weighted by uncertainty at levels 1 and 2, and precision-weighted contingency PE (**ε**_**3**_) by uncertainty at levels 2 and 3. The punctate change points contained in the true generative process are detected implicitly by the HGF via spikes in the precision weights. At levels 2 and 3, $\alpha_{i}^{\left(t\right)}$ is proportional to the precision ratio, $\psi_{i}^{\left(t\right)}$, defined in Eq 3. At level 1, the learning rate ***α***_1_ is simply defined as the update divided by the PE:

$$\alpha_{1}^{\left(t\right)}\propto \;\frac{\mu_{2}^{\left(t\right)}-{\hat{\mu }}_{1}^{\left(t\right)}}{\delta_{1}^{\left(t\right)}}\;.$$ (4)

Two participant-specific perceptual parameters, ϑ and ω, couple the hierarchical levels and allow for individual expression of approximate Bayes-optimal learning. ϑ determines the speed of learning about the volatility of the environment, i.e., the rate at which estimates of trialwise phasic volatility (μ_3_) are updated. Higher ϑ values imply a belief in a more unstable world and lead to a more variable learning rate. By contrast, ω is a constant component of the volatility and captures a tonic learning rate about stimulus transition contingencies (see Fig 3A and S1 Text for details).

#### Response model

The response model describes the mapping from a participant’s trialwise beliefs, as provided by the perceptual model, onto his/her observed responses, log(RTs). We reasoned that there are several variables that could influence trialwise log(RT). Therefore, we constructed and compared three response models using random effects Bayesian model comparison [108,109] and associated techniques for assessing differences in model frequencies across groups as implemented in the VBA toolbox [110]. Each model proposed that log(RT) on any given trial is a linear function of a constant component of log(RT) and several other factors. Since there is evidence, both from earlier work [80–83] and the present study, that participants’ RTs increase on a trial following an incorrect response, we included post-error slowing in each response model. While the perceptual model assumed that participants updated their beliefs according to the stimulus presented on each trial, the response model incorporated correct trials only.

The extra factors in the different models came from quantities at each level of the HGF that might influence log(RT). The first response model contained the following parameters: δ_1_ (sensory PE), because of evidence that DA sensitises motor responses to low-level PE [58,59]; ε_3_ (precision-weighted contingency PE), which has been shown to correlate with activity in the cholinergic basal forebrain [15]; and μ_3_ (estimated phasic volatility), which is relevant to cognitive switching tasks for which there is a proposed role for DA. For each parameter, the quantity relates to the stimulus transition that actually occurred on each trial. ζ is Gaussian noise.

*Response Model 1:*

$$\text{log}{\left(\text{RT}\right)}^{\left(\text{t}\right)}={\;\beta }_{0}+{\;\beta }_{1}(\delta_{1}^{\left(\text{t}\right)})\;+{\;\beta }_{2}(\varepsilon_{3}^{\left(\text{t}\right)})\;+{\;\beta }_{3}(\mu_{3}^{\left(\text{t}\right)})\;+{\;\beta }_{4}({\text{PostError}}^{\left(\text{t}\right)})\;+{\;\zeta }^{\left(\text{t}\right)}$$ (5)

Alternative research has indicated that activity in the dopaminergic midbrain correlates with the precision-weighted form of sensory PE, ε_2_ [15]. To disambiguate whether motor responses are modulated according to raw sensory PE or the confidence one has in their sensory predictions, our second model contained ε_2_ instead of δ_1_.

*Response Model 2:*

$$\text{log}{\left(\text{RT}\right)}^{\left(\text{t}\right)}={\;\beta }_{0}+{\;\beta }_{1}(\varepsilon_{2}^{\left(\text{t}\right)})\;+{\;\beta }_{2}(\varepsilon_{3}^{\left(\text{t}\right)})\;+{\;\beta }_{3}(\mu_{3}^{\left(\text{t}\right)})\;+{\;\beta }_{4}({\text{PostError}}^{\left(\text{t}\right)})\;+{\;\zeta }^{\left(\text{t}\right)}$$ (6)

Since δ_1_ and ε_2_ are highly correlated, we also constructed a third response model containing both parameters so as to ascertain whether one had a higher degree of explanatory power in terms of determining log(RT).

*Response Model 3:*

$$\text{log}{\left(\text{RT}\right)}^{\left(\text{t}\right)}={\;\beta }_{0}+{\;\beta }_{1}(\delta_{1}^{\left(\text{t}\right)})\;+{\;\beta }_{2}(\varepsilon_{2}^{\left(\text{t}\right)})\;+\beta_{3}(\varepsilon_{3}^{\left(\text{t}\right)})\;+{\;\beta }_{4}(\mu_{3}^{\left(\text{t}\right)})\;+{\;\beta }_{5}({\text{PostError}}^{\left(\text{t}\right)})\;+{\;\zeta }^{\left(\text{t}\right)}$$ (7)

**Fig 7:**
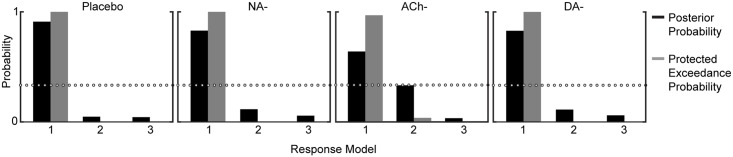
Random effects Bayesian model comparison confirmed that Response Model 1 was superior in all four groups. Posterior probabilities quantify the likelihood of each model given the data. Protected exceedance probabilities quantify how likely it is that any given model is more frequent than all other models in the comparison set while also protecting against the possibility that the observed variability in (log-) model evidences could be due to chance. The dotted line indicates the threshold for chance-level posterior probabilities (0.33). http://dx.doi.org/10.6084/m9.figshare.3796413.v1.

Random effects Bayesian model comparison established that Response Model 1 (containing parameters δ_1_, ε_3_, and μ_3_) was superior in all four pharmacological groups by a considerable margin: for the Placebo, NA-, ACh-, and DA- groups respectively, the posterior model probabilities were 0.911, 0.828, 0.636, and 0.829; protected exceedance probabilities (i.e., the probability that Response Model 1 is more likely than any other model in the comparison set) were 1.000, 1.000, 0.964, and 1.000 (Fig 7). Moreover, no significant difference in model frequencies between the Placebo group and any of the drug groups was identified (NA- versus Placebo: *p* = 0.958, ACh- versus Placebo: *p* = 0.560, DA- versus Placebo: *p* = 0.955). To further verify that Response Model 1 offered the best means by which to explain trialwise log(RT), we also compared a more exhaustive set of models containing different combinations of parameters from the HGF (see S3 and S4 Tables for details). Again, Response Model 1 was found to be superior and was therefore used for all subsequent analyses.

#### Model fitting

For each participant, individual maximum a posteriori estimates for perceptual and response model parameters were jointly obtained using the Broyden-Fletcher-Goldfarb-Shanno algorithm as implemented in the HGF Toolbox. Where priors were required, we defined them by inverting the perceptual model in isolation, given the known stimulus sequence (using the function “tapas_bayes_optimal_whatworld_config”), under suitably uninformative priors. We then used the resulting posterior estimates to define our priors for the subsequent inversion of the full model given the behavioural data (see S1 Table). In other words, the prior means in our empirical data analysis corresponded to those parameter values for which our stimulus sequence would generate minimal surprise (in an observer with the aforementioned uninformative priors).

Our key question pertained to how different neuromodulators influence learning and response modulation. Therefore, to make planned comparisons between the three active drug groups (NA-, ACh-, and DA-) and the Placebo group, we fit linear models separately for each participant-specific parameter (ϑ, ω, and each β, respectively), with additional covariates (body weight and Δalertness). For pairwise comparisons, Cohen’s *d* was used as a measure of effect size. In Fig 6, we present the results of the uncorrected, planned pairwise comparisons taken from each linear model and those that survive a Benjamini-Hochberg correction for three pairwise comparisons, i.e., between each drug group and Placebo.

### Control Analyses

We validated our model parameter results using permutation tests. Moreover, to further illustrate the implications of different model parameters and to demonstrate that the HGF provided a good fit to the behavioural data, we assessed the correlations between model parameters, analysed residuals, and used the HGF to generate simulated log(RT) data. Please see S1 Text for details.

## Supporting Information

S1 Fig Probabilistic structure of the experimental task. At any given time, the trial sequence was generated by one of eight TMs, which changed every 50 trials. In each case, there were 16 combinations that determined the probabilistic relationship between the stimuli presented on the current trial, *t*, and the previous trial, *t-1*. TMs 1 and 2 generated first-order stimulus sequences in which there was a high probability of the sequences 1-2-3-4 and 4-3-2-1 occurring, respectively. TMs 3 and 4 resulted in a high probability of alternating between two stimuli. TMs 5–8 were zeroth-order and led to one stimulus occurring with a high probability, one stimulus with a mid-range probability, and two stimuli with a low probability. Over the course of the experiment, each of the TMs occurred three times in a pseudorandom order, with no consecutive repeats. The overall probability of each stimulus was equal across 1,200 trials.
(TIF)

S2 Fig Model parameter correlations. Unless stated otherwise, Bayesian parameter averages (BPAs) and posterior means for the model parameters were only moderately correlated across groups (all absolute r < 0.660 for BPAs and r < 0.716 for posterior means). Higher correlations existed between BPAs for ω (transition contingency learning rate) and μ_3__0 (the initial phasic volatility estimate) (r = −0.948, −0.764, −0.771, and −0.983 for Placebo, NA-, ACh-, and DA-, respectively). Higher correlations also occurred between the BPAs (r = −0.877, −0.736, −0.630, and −0.880) and mean posteriors (r = -0.938, -0.947, -0.873, and -0.893) for β_0_ (log(RT) constant) and β_3_(μ_3_) (the sensitivity of log(RTs) to phasic volatility estimates). σ_3__0 is the initial value of σ_3_ (the uncertainty about the phasic volatility estimate). http://dx.doi.org/10.6084/m9.figshare.3796434.v1.
(TIF)

S3 Fig Residuals between observed log(RTs) and log(RTs) predicted by the HGF. The distribution of residuals suggests that, across drug groups, the model captured any patterns in the data well. Data are mean ± SEM. http://dx.doi.org/10.6084/m9.figshare.3796443.v1.
(TIF)

S4 Fig Autocorrelation between residuals across trials indicate that the model did not systematically under- or overestimate log(RTs) at true change points. Data are mean ± SEM. http://dx.doi.org/10.6084/m9.figshare.3796446.v1.
(TIF)

S5 Fig Mean log(RTs) for empirical and simulated data. Empirical data (filled bars) indicated that log(RT) increased as a stimulus’ true transition probability decreased. Simulated data (unfilled bars) generated using the posteriors for each participant in the Placebo group as model parameters faithfully reflect the empirical Placebo data. By shifting the parameters significantly altered by our different drug manipulations by the difference between the Placebo group mean for those parameters and the relevant drug-group mean, we simulated log(RT) data comparable to the empirical data observed in each drug-group. Note that there are no post-error slowing effects in the simulated data. Data are mean ± SEM. http://dx.doi.org/10.6084/m9.figshare.3796449.v2
(TIF)

S6 Fig Responses to high probability events become faster with learning but are slower after change points. (A) Mean baseline-corrected RTs for the first 25 (High-probability) trials in each context, where the baseline is the mean RT of the last three High-probability trials in the previous context. RTs increase following a true change point but fall as participants learn the new contextual rule. (B) As in A, but for simulated RTs. The model neatly captures the increase in RTs following true change points, the reduction in RT that occurs with learning across the course of the new context, and the suppressed effect of both in the ACh- group. http://dx.doi.org/10.6084/m9.figshare.3796452.v2.
(TIF)

S1 Table A summary of HGF parameters and priors. All priors are specified in the space in which they are estimated. For an account of how this relates to the native space of that parameter, the reader is referred to the original description of the model [9].
(DOCX)

S2 Table Placebo group averages for the perceptual and response model parameters. All results are mean ± SEM, corrected for the covariates Δalertness and body weight. β_0_ reflects a constant component of log(RT). β_1–4_ reflect the influence of sensory PE (δ_1_), precision-weighted contingency PE (ε_3_), phasic volatility estimates (μ_3_), and post-error trials on log(RT). All β values were significantly greater than zero (all *p* < 0.05), indicating that these parameters slowed log(RT). http://dx.doi.org/10.6084/m9.figshare.3796407.v1.
(DOCX)

S3 TableResults of familywise Bayesian model comparison.To further verify that Response Model 1 offered the best means by which to explain trialwise log(RT), we compared a more exhaustive set of linear response models containing different combinations of parameters from the HGF on the Placebo group. We first ran a familywise model comparison on models containing every combination of the parameters *δ*_*1*_, *ε*_*2*_, *ε*_*3*_, and *μ*_*3*_ (Family 1) versus models containing every combination of ${\hat{\sigma }}_{1}$, ${\hat{\sigma }}_{2}$, and ${\hat{\sigma }}_{3}$ (Family 2). Note that the quantities corresponded to the transition that actually occurred on each trial. All models included post-error slowing. Family 1 was found to be superior (posterior probability: 0.700; exceedance probability: 0.999).(DOCX)

S4 Table Results of exhaustive random effects Bayesian model comparison for models containing each combination of parameters δ_1_, ε_2_, ε_3_, and μ_3_. We ran random effects Bayesian model comparison on all the models in Family 1. Response Model 1 was found to be superior (posterior probability: 0.270; protected exceedance probability: 0.844).
(DOCX)

S5 Table Results of the permutation tests, randomising over drug assignment, for the HGF model parameters. Aside from the effect of ACh- on β_2_, all significant effects observed in the uncorrected multiple comparisons (cf. Fig 6) are mirrored in the results of the permutation tests. * *p* < 0.05, ** *p* < 0.01, *** *p* < 0.001; ns = nonsignificant.
(DOCX)

S1 Text Supporting information on physiological and subjective control measures, additional model-agnostic analyses, the HGF model, permutation tests, model parameter correlations, residuals, and simulations. (DOCX)

## Abbreviations

ACh: acetylcholine
BIS-11: Barratt Impulsiveness Scale
BOLD: blood-oxygen-level-dependent
BP: blood pressure
BPA: Bayesian parameter average
CFQ: Cognitive Failures Questionnaire
DA: dopamine
DOSPERT: Doman-Specific Risk Taking
HGF: Hierarchical Gaussian Filter
HR: heart rate
ITI: intertrial interval
LC: locus coeruleus
NA: noradrenaline
ns: nonsignificant
PE: prediction error
RM-ANOVA: repeated-measures analysis of variance
RT: reaction time
SD: standard deviation
SED: standard error of the difference
SEM: standard error of the mean
TM: transition matrix
VAS: visual analogue scale
YD: Yu and Dayan
