# Supplementary figures and images for: Pharmacological Fingerprints of Contextual Uncertainty
Source: PLoS Biol. 2016 Nov 15;14(11):e1002575. doi: 10.1371/journal.pbio.1002575 (PMC5113004; doi:10.1371/journal.pbio.1002575)

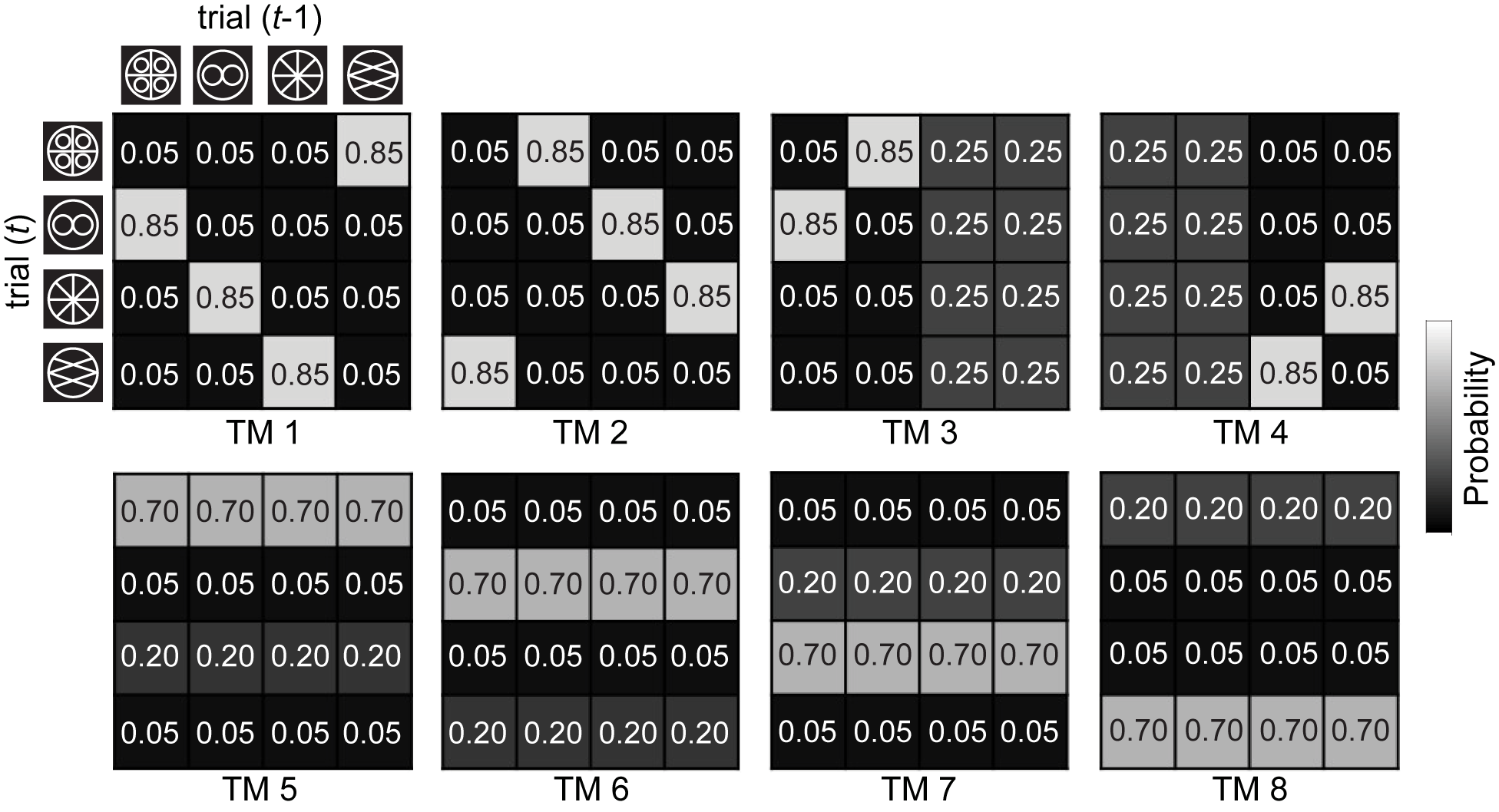

Supplement: S1 Fig — At any given time, the trial sequence was generated by one of eight TMs, which changed every 50 trials. In each case, there were 16 combinations that determined the probabilistic relationship between the stimuli presented on the current trial, t, and the previous trial, t-1. TMs 1 and 2 generated first-order stimulus sequences in which there was a high probability of the sequences 1-2-3-4 and 4-3-2-1 occurring, respectively. TMs 3 and 4 resulted in a high probability of alternating between two stimuli. TMs 5–8 were zeroth-order and led to one stimulus occurring with a high probability, one stimulus with a mid-range probability, and two stimuli with a low probability. Over the course of the experiment, each of the TMs occurred three times in a pseudorandom order, with no consecutive repeats. The overall probability of each stimulus was equal across 1,200 trials. (TIF) [file pbio.1002575.s001.tif]

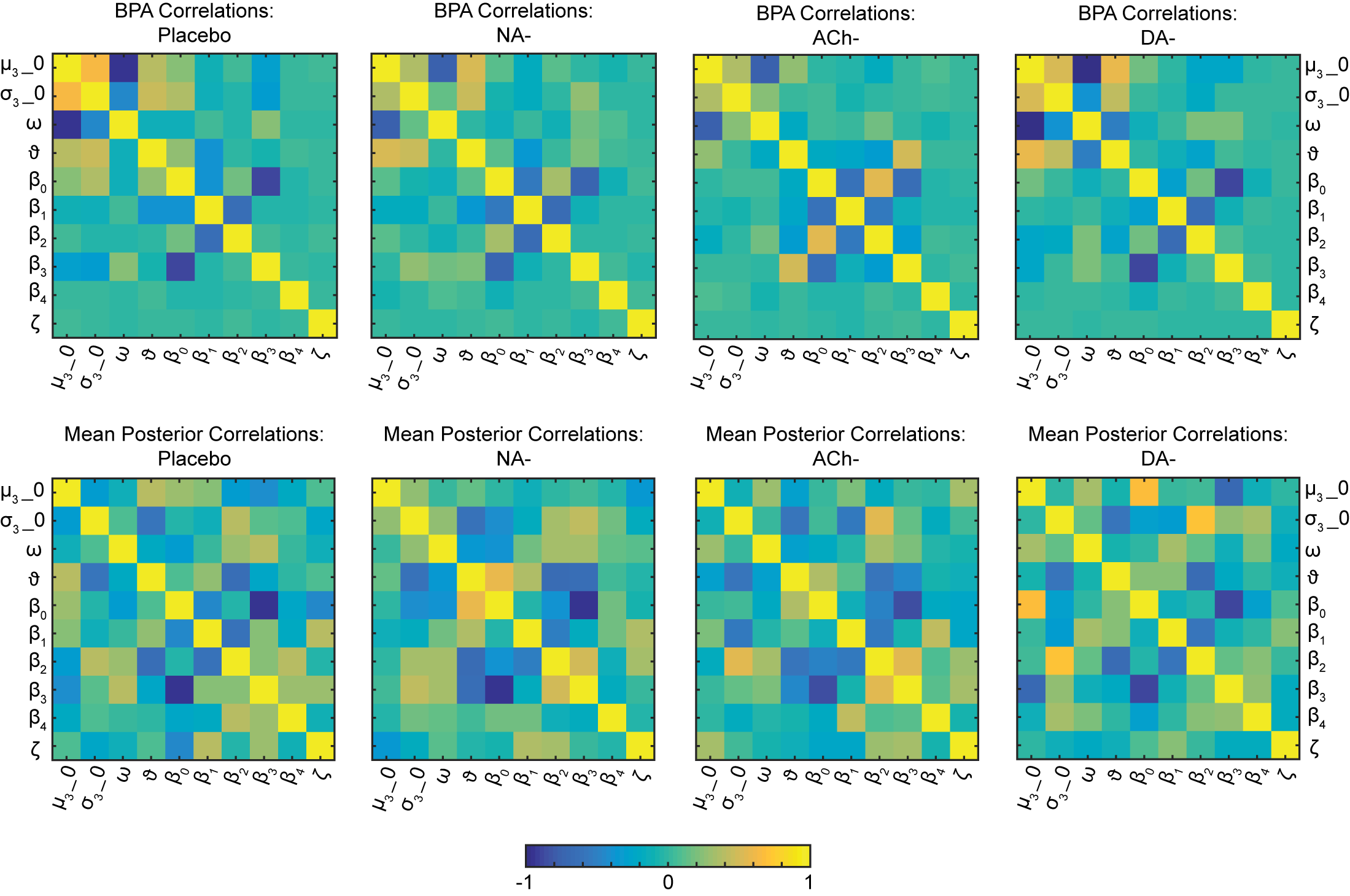

Supplement: S2 Fig — Unless stated otherwise, Bayesian parameter averages (BPAs) and posterior means for the model parameters were only moderately correlated across groups (all absolute r < 0.660 for BPAs and r < 0.716 for posterior means). Higher correlations existed between BPAs for ω (transition contingency learning rate) and μ3_0 (the initial phasic volatility estimate) (r = −0.948, −0.764, −0.771, and −0.983 for Placebo, NA-, ACh-, and DA-, respectively). Higher correlations also occurred between the BPAs (r = −0.877, −0.736, −0.630, and −0.880) and mean posteriors (r = -0.938, -0.947, -0.873, and -0.893) for β0 (log(RT) constant) and β3(μ3) (the sensitivity of log(RTs) to phasic volatility estimates). σ3_0 is the initial value of σ3 (the uncertainty about the phasic volatility estimate). http://dx.doi.org/10.6084/m9.figshare.3796434.v1. (TIF) [file pbio.1002575.s002.tif]

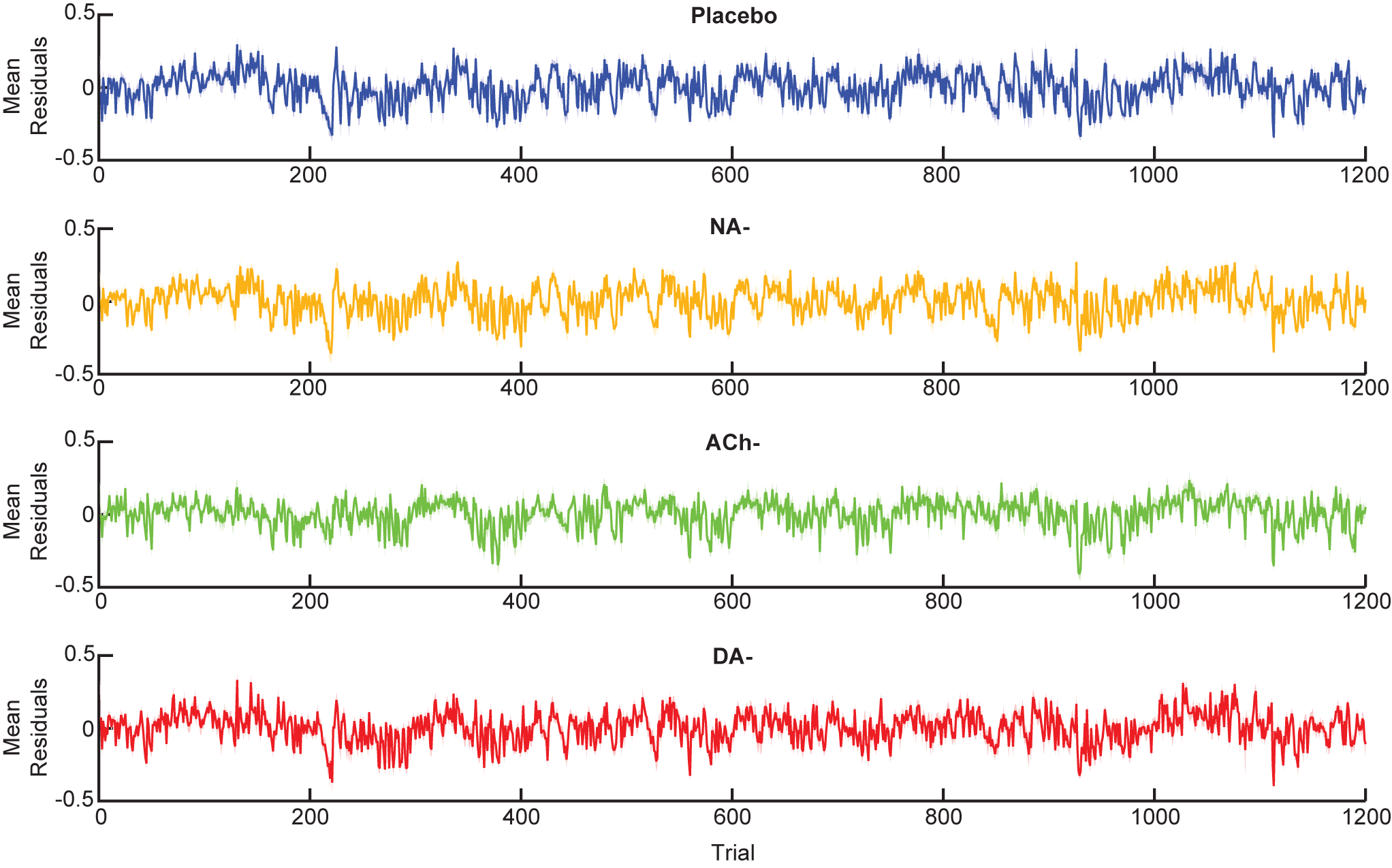

Supplement: S3 Fig — The distribution of residuals suggests that, across drug groups, the model captured any patterns in the data well. Data are mean ± SEM. http://dx.doi.org/10.6084/m9.figshare.3796443.v1. (TIF) [file pbio.1002575.s003.tif]

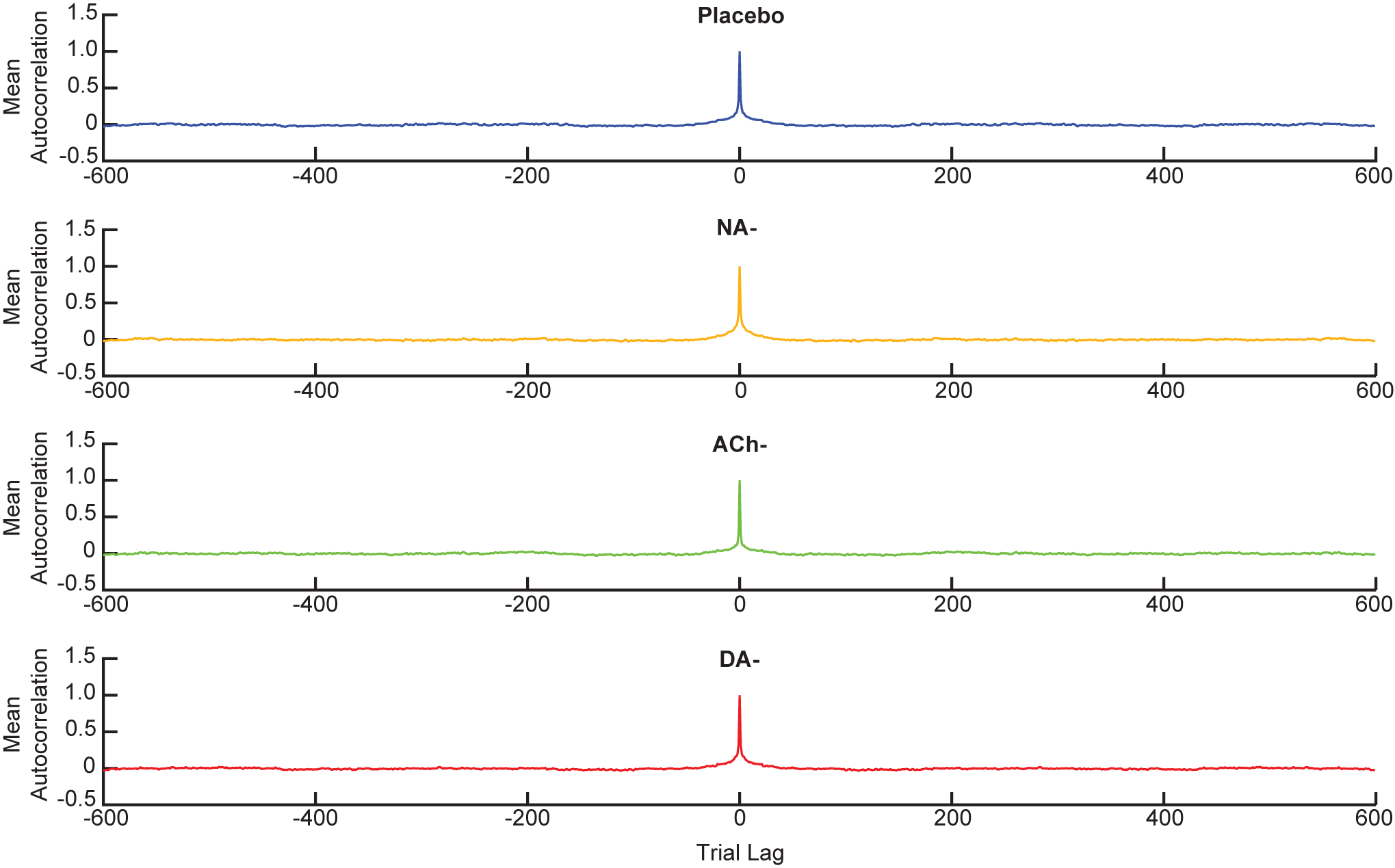

Supplement: S4 Fig — http://dx.doi.org/10.6084/m9.figshare.3796446.v1. (TIF) [file pbio.1002575.s004.tif]

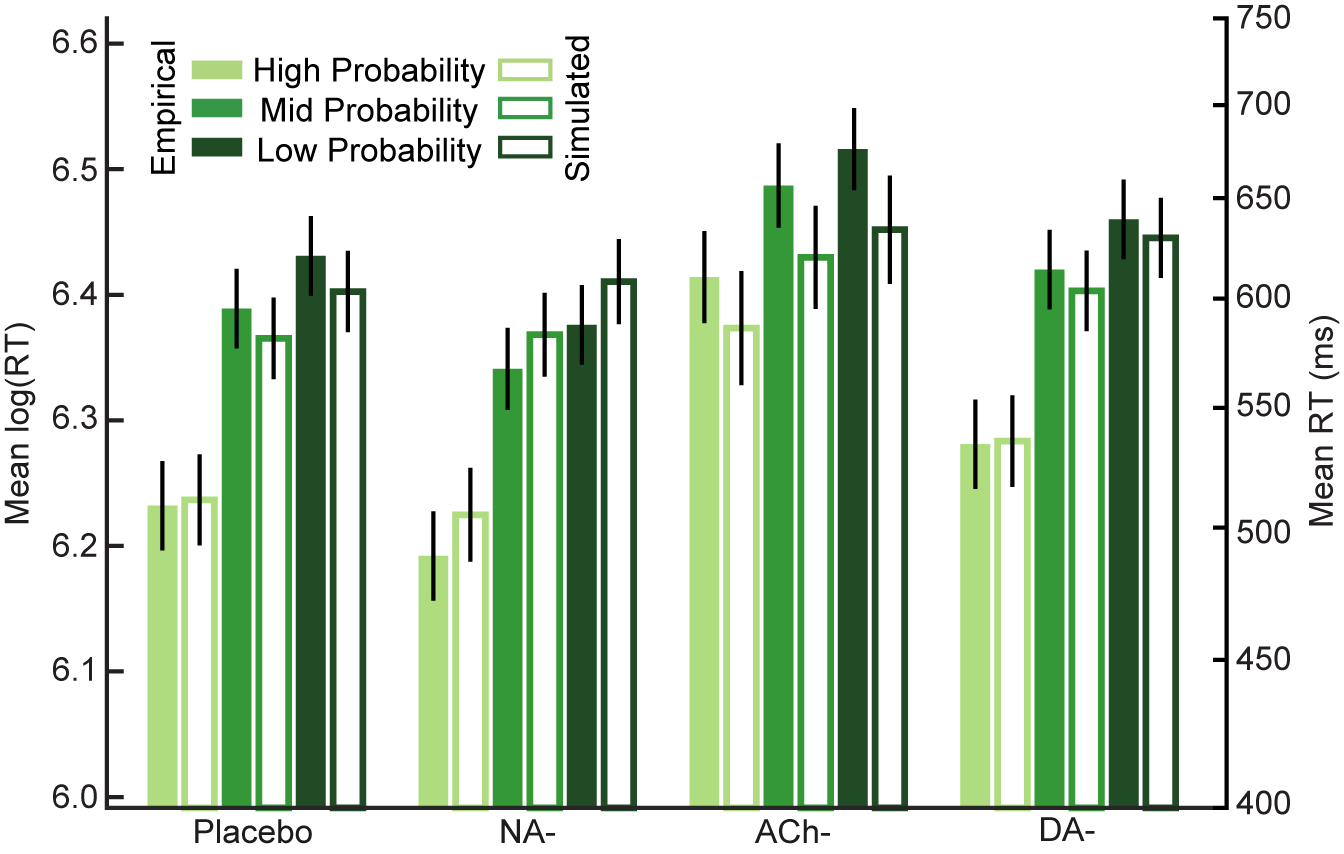

Supplement: S5 Fig — Empirical data (filled bars) indicated that log(RT) increased as a stimulus’ true transition probability decreased. Simulated data (unfilled bars) generated using the posteriors for each participant in the Placebo group as model parameters faithfully reflect the empirical Placebo data. By shifting the parameters significantly altered by our different drug manipulations by the difference between the Placebo group mean for those parameters and the relevant drug-group mean, we simulated log(RT) data comparable to the empirical data observed in each drug-group. Note that there are no post-error slowing effects in the simulated data. Data are mean ± SEM. http://dx.doi.org/10.6084/m9.figshare.3796449.v2 (TIF) [file pbio.1002575.s005.tif]

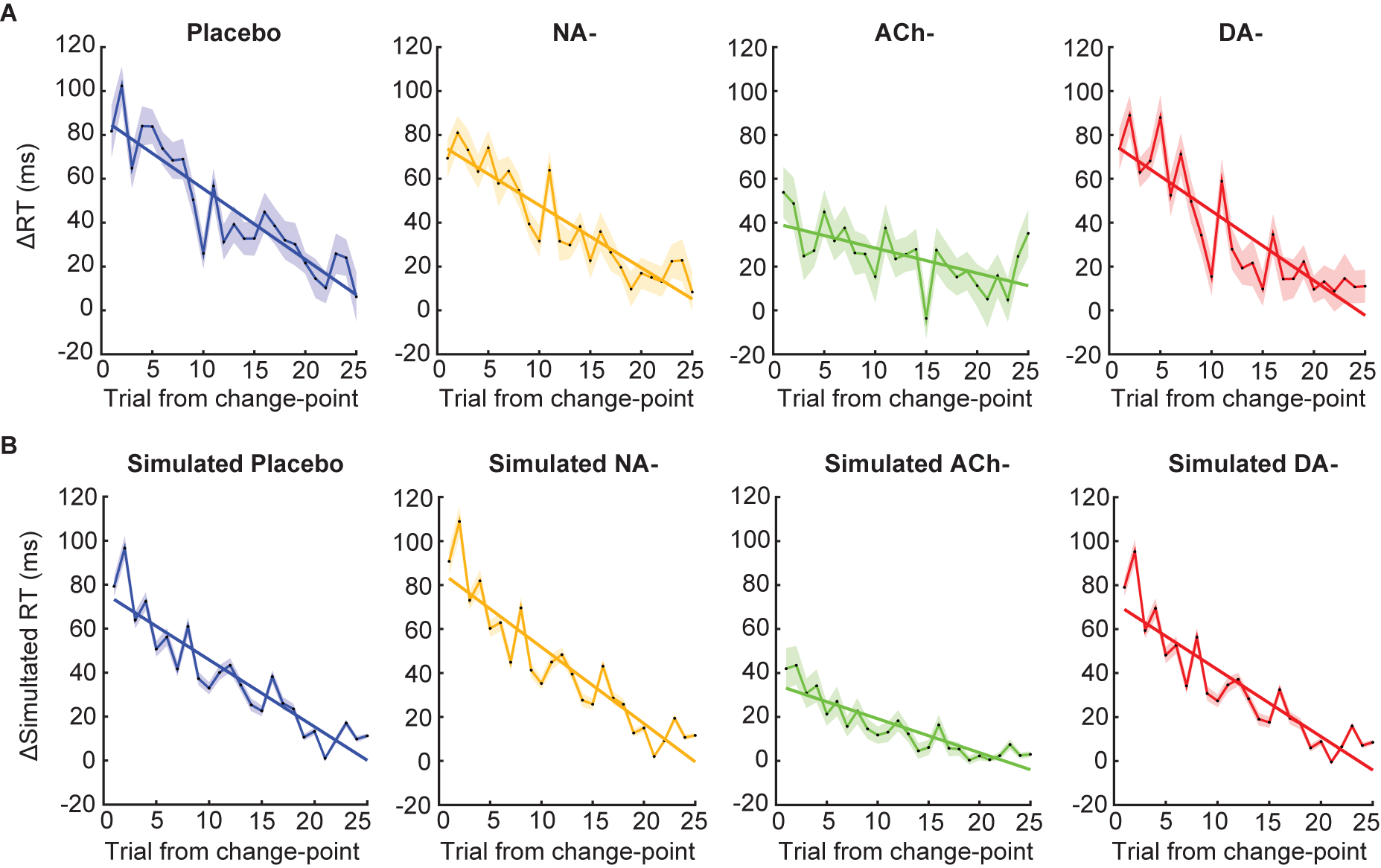

Supplement: S6 Fig — (A) Mean baseline-corrected RTs for the first 25 (High-probability) trials in each context, where the baseline is the mean RT of the last three High-probability trials in the previous context. RTs increase following a true change point but fall as participants learn the new contextual rule. (B) As in A, but for simulated RTs. The model neatly captures the increase in RTs following true change points, the reduction in RT that occurs with learning across the course of the new context, and the suppressed effect of both in the ACh- group. http://dx.doi.org/10.6084/m9.figshare.3796452.v2. (TIF) [file pbio.1002575.s006.tif]
