# Supplementary material for: Pharmacological Fingerprints of Contextual Uncertainty
Source: PLoS Biol. 2016 Nov 15;14(11):e1002575. doi: 10.1371/journal.pbio.1002575 (PMC5113004; doi:10.1371/journal.pbio.1002575)
Supplement: S1 Table — All priors are specified in the space in which they are estimated. For an account of how this relates to the native space of that parameter, the reader is referred to the original description of the model [9]. (DOCX) [file pbio.1002575.s007.docx]

| **Parameter** | **Notes** | **Prior** | | |
| --- | --- | --- | --- | --- |
| **Perceptual Model** | | | | |
| ϑ | Metavolatility belief parameter; controls the step size of the Gaussian random walk at level 3. Estimated in logit space. | Mean  Variance  Upper bound | | 0  2  0.01 |
| ω | Tonic volatility belief parameter; a constant component of the learning rate at level 2. | Mean  Variance | | -6  25 |
| Stimulus Transitions  (**x**_1_) | 4x4 matrix; the predictions are a sigmoid transformation of the probabilities represented in **x**_2_, and so do not have a starting value. | **μ**_1_**_:_**  Mean  Variance | NaN  NaN | |
|  |  | **σ**_1_**_:_**  Mean  Variance | NaN  NaN | |
| Stimulus Transition Contingencies  (**x**_2_) | 4x4 matrix; estimated conditional probabilities for each of the 16 possible stimulus transitions are updated on each trial. At level 2, estimates are made in logit space (-1.0986 is equivalent to a probability of 0.25). | **μ**_2_**_:_**  Mean  Variance | -1.0986  0 | |
|  |  | **σ**_2_**_:_**  Mean  Variance | 0  log(1) | |
| Volatility  (x_3_) | Scalar; one trial-wise volatility estimate is updated after each stimulus transition. | μ_3:_  Mean  Variance | 1  0.1 | |
|  |  | σ_3:_  Mean  Variance | log(0.1)  1 | |
| **Response Model** *(captures the influence of various parameters on log(RT))* | | | | |
| β_0_ | log(RT) constant | Mean  Variance | log(500)  3 | |
| β_1_ | Sensory PE (δ_1_) | Mean  Variance | 0  4 | |
| β_2_ | Precision-weighted contingency PE (ε_3_) | Mean  Variance | 0  4 | |
| β_3_ | Volatility estimate (μ_3_) | Mean  Variance | 0  4 | |
| β_4_ | Post-error | Mean  Variance | 0  3 | |
| ζ | Noise | Mean  Variance | -3  1e^-3^ | |
