# Supplementary material for: Pharmacological Fingerprints of Contextual Uncertainty
Source: PLoS Biol. 2016 Nov 15;14(11):e1002575. doi: 10.1371/journal.pbio.1002575 (PMC5113004; doi:10.1371/journal.pbio.1002575)
Supplement: S2 Table — All results are mean ± SEM, corrected for the covariates Δalertness and body weight. β0 reflects a constant component of log(RT). β1–4 reflect the influence of sensory PE (δ1), precision-weighted contingency PE (ε3), phasic volatility estimates (μ3), and post-error trials on log(RT). All β values were significantly greater than zero (all p < 0.05), indicating that these parameters slowed log(RT). http://dx.doi.org/10.6084/m9.figshare.3796407.v1. (DOCX) [file pbio.1002575.s008.docx]

| **Parameter** | **Mean** | **SEM** | **t-value** | **p-value** |
| --- | --- | --- | --- | --- |
| ϑ | 0.0021 | 0.0010 | - | - |
| ω | -2.8654 | 0.5503 | - | - |
| β_0_ | 5.5108 | 0.2919 | 18.879 | <0.001 |
| β_1_(δ_1_) | 0.1960 | 0.0745 | 2.631 | 0.010 |
| β_2_(ε_3_) | 0.3893 | 0.1040 | 3.744 | <0.001 |
| β_3_(μ_3_) | 0.5727 | 0.2799 | 2.046 | 0.043 |
| β_4_(Post-Error) | 0.0960 | 0.0311 | 3.086 | 0.003 |
| ζ | 0.0527 | 0.0038 | - | - |
