# Supplementary material for: Pharmacological Fingerprints of Contextual Uncertainty
Source: PLoS Biol. 2016 Nov 15;14(11):e1002575. doi: 10.1371/journal.pbio.1002575 (PMC5113004; doi:10.1371/journal.pbio.1002575)
Supplement: S3 Table — To further verify that Response Model 1 offered the best means by which to explain trialwise log(RT), we compared a more exhaustive set of linear response models containing different combinations of parameters from the HGF on the Placebo group. We first ran a familywise model comparison on models containing every combination of the parameters δ1, ε2, ε3, and μ3 (Family 1) versus models containing every combination of σ^1, σ^2, and σ^3 (Family 2). Note that the quantities corresponded to the transition that actually occurred on each trial. All models included post-error slowing. Family 1 was found to be superior (posterior probability: 0.700; exceedance probability: 0.999). (DOCX) [file pbio.1002575.s009.docx]

|  | **Model**  **Parameters** | **Posterior Probability** | **Exceedance Probability** |
| --- | --- | --- | --- |
| **Family 1** | δ_1_, ε_3_, μ_3_, PostError, ζ  ε_2_, ε_3_, μ_3_, PostError, ζ  δ_1_, ε_2_, ε_3_, μ_3_, PostError, ζ  δ_1_, PostError, ζ  δ_1_, ε_2_, PostError, ζ  δ_1_, ε_2_, ε_3_, PostError, ζ  δ_1_, ε_2_, μ_3_, PostError, ζ  δ_1_, ε_3_, PostError, ζ  δ_1_, μ_3_, PostError, ζ  ε_2_ PostError, ζ  ε_2_, ε_3_, PostError, ζ  ε_2_, μ_3_, PostError, ζ  ε_3_, PostError, ζ  ε_3_, μ_3_, PostError, ζ  μ_3_, PostError, ζ | 0.700 | 0.999 |
| **Family 2** | $\hat{\sigma}_{1}$, PostError, ζ  $\hat{\sigma}_{2}$, PostError, ζ  $\hat{\sigma}_{3}$, PostError, ζ  $\hat{\sigma}_{1}, \hat{\sigma}_{2}$, PostError, ζ  $\hat{\sigma}_{2}, \hat{\sigma}_{3}$, PostError, ζ  $\hat{\sigma}_{1}, \hat{\sigma}_{2}$, $\hat{\sigma}_{3}$, PostError, ζ | 0.300 | 0.001 |
