# Supplementary material for: Pharmacological Fingerprints of Contextual Uncertainty
Source: PLoS Biol. 2016 Nov 15;14(11):e1002575. doi: 10.1371/journal.pbio.1002575 (PMC5113004; doi:10.1371/journal.pbio.1002575)
Supplement: S4 Table — We ran random effects Bayesian model comparison on all the models in Family 1. Response Model 1 was found to be superior (posterior probability: 0.270; protected exceedance probability: 0.844). (DOCX) [file pbio.1002575.s010.docx]

| **Model**  **Number** | **Model**  **Parameters** | **Posterior**  **Probability** | **Protected Exceedance Probability** |
| --- | --- | --- | --- |
| 1 | δ_1_, ε_3_, μ_3_, PostError, ζ | 0.270 | 0.844 |
| 2 | ε_2_, ε_3_, μ_3_, PostError, ζ | 0.023 | 0.000 |
| 3 | δ_1_, ε_2_, ε_3_, μ_3_, PostError, ζ | 0.024 | 0.000 |
| 4 | δ_1_, PostError, ζ | 0.115 | 0.026 |
| 5 | δ_1_, ε_2_, PostError, ζ | 0.023 | 0.000 |
| 6 | δ_1_, ε_2_, ε_3_, PostError, ζ | 0.022 | 0.000 |
| 7 | δ_1_, ε_2_, μ_3_, PostError, ζ | 0.022 | 0.000 |
| 8 | δ_1_, ε_3_, PostError, ζ | 0.024 | 0.000 |
| 9 | δ_1_, μ_3_, PostError, ζ | 0.144 | 0.068 |
| 10 | ε_2_ PostError, ζ | 0.023 | 0.000 |
| 11 | ε_2_, ε_3_, PostError, ζ | 0.038 | 0.000 |
| 12 | ε_2_, μ_3_, PostError, ζ | 0.021 | 0.000 |
| 13 | ε_3_, PostError, ζ | 0.097 | 0.013 |
| 14 | ε_3_, μ_3_, PostError, ζ | 0.133 | 0.049 |
| 15 | μ_3_, PostError, ζ | 0.021 | 0.000 |
