# Supplementary material for: Pharmacological Fingerprints of Contextual Uncertainty
Source: PLoS Biol. 2016 Nov 15;14(11):e1002575. doi: 10.1371/journal.pbio.1002575 (PMC5113004; doi:10.1371/journal.pbio.1002575)
Supplement: S5 Table — Aside from the effect of ACh- on β2, all significant effects observed in the uncorrected multiple comparisons (cf. Fig 6) are mirrored in the results of the permutation tests. * p < 0.05, ** p < 0.01, *** p < 0.001; ns = nonsignificant. (DOCX) [file pbio.1002575.s011.docx]

|  | **Comparison**  **(Drug vs Placebo)** | **Direction of effect**  **(Drug vs Placebo)** | **p-value** | **Significant?** |
| --- | --- | --- | --- | --- |
| **ϑ** | NA- vs Placebo | ↑ | 0.040 | * |
|  | ACh- vs Placebo | ↑ | 0.016 | * |
|  | DA- vs Placebo | - | 0.147 | ns |
| **ω** | NA- vs Placebo | - | 0.203 | ns |
|  | ACh- vs Placebo | ↓ | 0.002 | ** |
|  | DA- vs Placebo | - | 00106 | ns |
| **β_0_** | NA- vs Placebo | - | 0.322 | ns |
|  | ACh- vs Placebo | ↑ | <0.001 | *** |
|  | DA- vs Placebo | ↑ | 0.011 | * |
| **β_1_** | NA- vs Placebo | - | 0.568 | ns |
|  | ACh- vs Placebo | ↓ | <0.001 | *** |
|  | DA- vs Placebo | - | 0.392 | ns |
| **β_2_** | NA- vs Placebo | - | 0.130 | ns |
|  | ACh- vs Placebo | - | 0.057 | ns |
|  | DA- vs Placebo | - | 0.104 | ns |
| **β_3_** | NA- vs Placebo | - | 0.122 | ns |
|  | ACh- vs Placebo | ↓ | <0.001 | *** |
|  | DA- vs Placebo | ↓ | 0.006 | ** |
| **β_4_** | NA- vs Placebo | - | 0.554 | ns |
|  | ACh- vs Placebo | - | 0.542 | ns |
|  | DA- vs Placebo | - | 0.711 | ns |
| **ζ** | NA- vs Placebo | - | 0.571 | ns |
|  | ACh- vs Placebo | - | 0.098 | ns |
|  | DA- vs Placebo | - | 0.505 | ns |
