# Supplementary material for: Pharmacological Fingerprints of Contextual Uncertainty
Source: PLoS Biol. 2016 Nov 15;14(11):e1002575. doi: 10.1371/journal.pbio.1002575 (PMC5113004; doi:10.1371/journal.pbio.1002575)
Supplement: S1 Text — (DOCX) [file pbio.1002575.s012.docx]

**Supporting Information: Pharmacological Fingerprints of Contextual Uncertainty**

Louise Marshall^#^, Christoph Mathys^#^, Diane Ruge, Archy O. de Berker, Peter Dayan, Klaas E. Stephan, Sven Bestmann

**Physiological and Subjective Control Measures**

Each of the four groups showed equivalent baseline working memory (Kruskal-Wallis test on discrete data: H_3_=4.09, p=0.252), impulsivity (one-way ANOVA on continuous data: F_3,120_=0.858, p=0.465), distractibility (F_3,120_=1.64, p=0.185) and risk-taking behaviour (F_3,120_=1.40, p=0.245). They also reported having received the same quantity (H_3_=2.30, p=0.513) and quality (H_3_=1.78, p=0.620) of sleep on the previous night and equivalently self-scored their fatigue during the reaction time (RT) task (F_3,120_=0.23, p=0.876). 50% of Placebo, 77% of NA-, 86% of ACh-, and 44% of DA- participants reported that they thought they had received an active drug (H_3_=16.85, p=0.001).

Ratings for alertness, calmness and contentedness all changed significantly over the course of the experiment (F_1.79,214.39_=71.60, p<0.001, η_p_^2^=0.374; F_1.88,225.25_=5.96, p=0.004, η_p_^2^=0.047 and F_2,240_=25.65, p<0.001, η_p_^2^=0.176 respectively), but only alertness ratings showed a significant time x drug interaction (F_5.36,214.39_=6.40, p<0.001, η_p_^2^=0.138). On average, alertness decreased within-participants over the course of the experiment in all four groups. A one-way ANOVA with drug as a between-subject factor revealed that the degree to which alertness decreased between baseline and the time corresponding to peak drug concentration varied between groups (F_3,120_=7.92, p<0.001, η_p_^2^=0.165). More specifically, compared to Placebo, the alertness decrease was significantly more pronounced in the ACh- and NA- groups (t=-4.31, p<0.001, *d=*-1.11 and t=-2.76, p=0.007, *d=*-0.70 respectively).

Heart rate (HR) varied significantly with time (F_1.89, 226.71_=129.25, p<0.001, η_p_^2^=0.519) and this effect was modulated by drug-group (F_5.67,226.71_=5.40, p<0.001, η_p_^2^=0.119). On average, all groups showed participant-specific HR decreases between pre-drug and post-drug administration. The magnitude of HR deceleration differed between groups (F_3,120_=6.65, p<0.001, η_p_^2^=0.143), but only in the ACh- group was HR deceleration more pronounced than Placebo (t=-3.14, p=0.002, *d=*-0.81). While systolic blood pressure (BP) varied with time (F_2,240_=7.12, p=0.001, η_p_^2^=0.056), there was no time x drug interaction (F_6,240_=1.55, p=0.162). Diastolic BP showed no main effect of time (F_2,240_=0.37, p=0.695), but there was a significant time x drug interaction (F_6,240_=3.52, p=0.002, η_p_^2^=0.081). More precisely, participant-specific differences in diastolic BP between pre-drug and post-drug administration varied significantly between groups (F_3,120_=5.11, p=0.002, η_p_^2^=0.113) due to a significant decrease in diastolic BP in the NA- group compared to the Placebo group (t=-3.49, p<0.001, *d=*-0.88). This is unsurprising given that the NA- drug administered (prazosin) is used clinically as an anti-hypertensive. Raw data for all physiological and subjective control measures can be found at http://dx.doi.org/10.6084/m9.figshare.3168682.v1.

**Hierarchical Gaussian Filter (HGF)**

For behavioural analysis, we applied a Hierarchical Gaussian Filter (HGF) that describes learning at multiple levels and allows for inference on an agent’s belief about the causes of its sensory inputs [1]. Here we implemented a three-level HGF, as summarised in Fig 3A. Level 1 of the HGF represents a sequence of environmental states $\boldsymbol{x}_{1}$ (here the presentation of one of four stimuli). Level 2 represents the transition contingency $\boldsymbol{x}_{2}$ (i.e., the conditional probability, in logit space, of the stimulus on trial *t* given the stimulus presented on trial *t-1*). Each of these hidden states is assumed to evolve as a Gaussian random walk, such that its variance depends on the state at the level above:

$$p\left( x_{1,jk} | x_{2,jk} \right)= s\left( x_{2,jk} \right)^{x_{1,jk}}\left( 1-s\left( x_{2,jk} \right) \right)^{1-x_{1,jk}}=\mathrm{Bernoulli}\left( x_{1,jk};s\left( x_{2,jk} \right) \right)$$

(Equation A)

$$p\left( x_{2,jk}^{\left( t \right)} | x_{2,jk}^{\left( t-1 \right)},x_{3}^{\left( t \right)} \right)= N\left( x_{2,jk}^{(t)}; x_{2,jk}^{(t-1)}, exp\left( x_{3}^{(t)}+\omega\right) \right)$$

(Equation B)

$$p\left( x_{3}^{\left( t \right)} | x_{3}^{\left( t-1 \right)},\vartheta\right)= N\left( x_{3}^{(t)}; x_{3}^{(t-1)}, \vartheta\right)$$

(Equation C)

where $x_{1,jk}$ and $x_{2,jk}$ (with j,k=1,…,4) are the elements of the level 1 transition matrix $\boldsymbol{x}_{1}$ and of the level 2 matrix $\boldsymbol{x}_{2}$ respectively, and $s$ is the logistic sigmoid function

$$s\left( x \right)= \frac{1}{1+exp(-x)}$$

(Equation D)

In Equations B and C, ϑ determines the speed of learning about the volatility of the environment and ω is a constant component of the learning rate at level 2. In our instantiation of the HGF, ϑ and ω are free parameters.

***Precision-Weighted PE at Level 2***

At level 2 of the HGF, the precision-weighted PE about stimulus outcome (termed $\boldsymbol{\varepsilon}_{2}$) serves to update the belief about $\boldsymbol{x}_{2}$ (the stimulus transition contingency in logit space):

$$\mu_{2,jk}^{\left( t \right)}- \mu_{2,jk}^{\left( t-1 \right)}= \psi_{2,jk}^{\left( t \right)}\delta_{1,jk}^{\left( t \right)}$$

$$= \varepsilon_{2,jk}^{(t)}$$

(Equation E)

Here, the PE is the difference between the actual and the predicted outcome on trial *t*:

$$\delta_{1,jk}^{\left( t \right)}= \mu_{1,jk}^{\left( t \right)}- \hat{\mu}_{1,jk}^{\left( t \right)}$$

(Equation F)

where the prediction $\hat{\mu}_{1}^{\left( t \right)}$ about stimulus outcome results from a sigmoidal transformation of the previous belief about the stimulus transition contingency $\boldsymbol{\mu}_{2}^{\left( t-1 \right)}$:

$$\hat{\mu}_{1,jk}^{\left( t \right)}=s(\mu_{2,jk}^{(t-1)})$$

(Equation G)

At level 3, the update of the belief about $x_{3}$ (environmental log-volatility) is proportional to the precision-weighted PE $\boldsymbol{\varepsilon}_{3}$

$$\mu_{3}^{\left( t \right)}- \mu_{3}^{\left( t-1 \right)}\boldsymbol{\propto}\psi_{3,jk}^{\left( t \right)}\delta_{2,jk}^{\left( t \right)}$$

$$= \frac{\hat{\pi}_{2,jk}^{\left( t \right)}}{\pi_{3}^{\left( t \right)}}\delta_{2,jk}^{\left( t \right)}$$

$$= \varepsilon_{3,jk}^{(t)}$$

(Equation H)

Here, the PE concerns the volatility of the stimulus transition contingency, or more precisely, the variance ratio of its estimates (in logit space) after and before observing the sensory input respectively:

$$\delta_{2,jk}^{(t)}= \frac{\sigma_{2,jk}^{(t)}+{(\mu_{2,jk}^{\left( t \right)}- \mu_{2,jk}^{(t-1)})}^{2}}{\sigma_{2,jk}^{(t-1)}+ e^{{\mu_{3}}^{\left( t-1 \right)+\omega}}} -1$$

(Equation I)

Detailed explanation of the nature of the PEs can be found in [1].

**Model Parameter Correlations**

Aside from two exceptions, Bayesian Parameter Averages (BPAs) for the model parameters were only moderately correlated (all absolute r<0.660). Higher correlations existed between ω and the initial value of μ_3_ (r=-0.948, -0.764, -0.771, -0.983 for Placebo, NA-, ACh- and DA- respectively). This is to be expected on theoretical grounds because the two parameters perform very similar functions in the generative model. Note that we estimated the initial value of μ_3_ as we used it as a predictor of log(RT) in the Response Model. However, when we fixed μ_3__0 there were no changes to any of the reported main effects. Higher correlations also occurred between β_0_ and β_3_(μ_3_) (r=-0.877, -0.736, -0.630 and -0.880 for Placebo, NA-, ACh- and DA- respectively). Here the negative correlations indicate that both the constant component of log(RT) and phasic volatility estimate had a similar slowing effect on log(RT). This reflects the fact that, while including μ_3_ as a predictor of log(RT) significantly improves model evidence, it is much less variable than the other predictors because volatility inevitably changes at a slower time scale than stimulus contingencies. Similarly, when we examined the correlations between the mean posterior estimates for each parameter, the only higher correlations found consistently across drug-groups were for β_0_ and β_3_(μ_3_) (r=-0.938, -0.947, -0.873 and -0.893). All remaining absolute correlations were r<0.652, 0.662, 0.580 and 0.716 for Placebo NA-, ACh- and DA- respectively. See S2 Fig for details on all correlation results.

**Analysis of the Residuals between the Observed log(RTs) and those Predicted by the HGF**

For further verification that the HGF model provided a good fit to the behavioural data, we assessed the residuals between the observed log(RTs) and those predicted by the model. The distribution of the residuals suggests that the model indeed captured the patterns in the data well (S3 Fig). Moreover, autocorrelations between residuals for participants in each drug-group indicate that the model did not systematically under- or over-estimate log(RTs) at true change-points (S4 Fig). The mean (± SEM) correlations between observed log(RTs) and predicted log(RTs) were 0.38 ± 0.02, 0.36 ± 0.02, 0.26 ± 0.01, and 0.36 ± 0.02 for Placebo, NA-, ACh- and DA- respectively.

**Simulations**

To demonstrate that the HGF can capture the effects we report in the paper, and to illustrate the implications of different model parameters further, we used the HGF to generate simulated log(RT) data. First, we ran 100 simulations for each set of posterior parameter values obtained for each participant in the Placebo group, generating 1200 log(RTs) for each run. We then averaged the simulated log(RTs) on High (p=0.85 or p=0.70), Mid (p=0.25 or p=0.20) and Low (p=0.05) probability trials, i.e., to mirror the model-agnostic analyses. For each of a series of further simulations, we took the same parameter settings but this time modified particular parameters of interest, identified based on our empirical observations. For these parameters, we shifted the estimated parameters for each Placebo participant by the difference between the Placebo group average and the relevant drug-group average for that parameter. Again, we ran 100 runs for each “computationally drugged” participant and averaged the simulated log(RTs) across three probability levels. This allowed us to assess the impact of different model parameters on log(RT), and to compare simulated log(RTs) to empirical data in each drug-group.

Simulating log(RTs) using the posteriors for each participant in the Placebo group as parameter values produced mean data that faithfully reflected the increase in log(RT) with decreasing stimulus transition probability that we observed in the Placebo group’s empirical data (S5 Fig). Next we adjusted parameters for each Placebo participant in line with the perceptual and response effects of our pharmacological NA, ACh and DA manipulations. When we simulated NA antagonism by increasing ϑ by the difference between the Placebo group ϑ average and the NA- group ϑ average (0.0010), we observed simulated log(RTs) comparable to those for the NA- group. The same was true when we simulated DA antagonism by simultaneously increasing β_0_ by 0.6695 and decreasing β_3_ by 0.5014; again these were the differences between the Placebo group average and the DA- group average for parameters significantly altered by our dopaminergic manipulation. Similarly, simulating ACh antagonism by increasing ϑ by 0.0013 and β_0_ by 0.6695, and decreasing ω by 0.7623, β_1_ by 0.1631, β_2_ by 0.0844 and β_3_ by 0.5014 produced slower simulated log(RTs) that faithfully reflected the empirical ACh- log(RT) data. Note that, unlike the empirical data, there is no additional slowing caused by post-error effects in the simulated data.

The simulated data is also able to capture the increase in RT observed in the empirical data following true change-points (S6 Fig), as well as the learning that occurs across trials within a stable context. The model-agnostic data shown in S6A Fig is for mean (±SEM) ΔRT for high-probability trials (p>=0.70, as defined in the relevant transition matrix (TM)) on which participants made a correct response, following true change-points for each of the drug-groups. We have averaged across all TM-types. The trial-wise ΔRT measure is the difference between RT on each post-change trial and the average of the last three high probability, correct trials in the previous context. We can clearly see that RTs increase across drug-groups on the trial following a true change-point (one-way ANOVA with Δalertness and body weight covariates: F_5,118_=6.07, p<0.001, η_p_^2^=0.21), with an additional between-subject effect of drug-group (F_3,118_=6.52, p<0.001, η_p_^2^=0.14). Post-hoc comparisons (Benjamini-Hochberg-corrected) demonstrate that this RT increase is significantly reduced in the ACh- group compared to the Placebo (t_57_=-3.95, p<0.001) group. This is in line with our assessment that individuals in the ACh- group showed poorer learning of the contextual transition contingencies.

Moreover, over the course of the context, we see a decrease in RT for high-probability trials, reflecting learning of the new context. When we use an ANOVA, with Δalertness and body weight as covariates, to compare the lines of best fit for each participant’s ΔRTs, we find that there is learning across the course of the contexts (reflected by the negative slopes; effect of slope: F_5,118_=6.07, p<0.001, η_p_^2^=0.21) and that this is modulated by drug-group (effect of drug: F_3,118_=7.73, p<0.001, η_p_^2^=0.16). Again, corrected post-hoc comparisons show that the ACh- group shows slower learning compared to the Placebo group (t_57_=4.04, p<0.001) group, in line with our finding that transition contingency learning rate (as reflected by model parameter ω) was reduced following ACh antagonism.

S6B Fig indicates that the simulated data results echo those for the model-agnostic results: there are equivalent between-group differences, most notably a reduction in the RT increase following a true context switch, as well as a reduced learning rate, for the ACh- group.

**Permutation Tests**

In addition to the linear models used to assess the effects of our three drug manipulations on the HGF model parameters, we conducted permutation tests, randomising drug assignment over participants, to make distribution-free comparisons. We ran 10,000 permutations per parameter. For each parameter and each permutation, we calculated the difference between the mean for each permuted drug and the mean for the permuted Placebo. We then tested the values for the actual permutation by calculating the fraction of the permutation points with larger absolute differences than, but in the same direction as, those differences we observed in our empirical data. The results echo what we report in the paper. Aside from the effect of ACh- on β_2_, all significant effects observed in the uncorrected multiple comparisons detailed in Fig 6 are reflected in the results of the permutation tests (S5 Fig).

**Supplemental References**

1. Mathys C, Daunizeau J, Friston KJ, Stephan KE. A Bayesian foundation for individual learning under uncertainty. Front Hum Neurosci. 2011;5: 39.
